# LDL-C target attainment and treatment costs in high-risk and very-high-risk patients with or without bempedoic acid: A Spanish cohort simulation

**DOI:** 10.1371/journal.pone.0353193

**Published:** 2026-08-14

**Authors:** Juan Cosin-Sales, Maria Reyes Abad-Sazatornil, José Antonio Martín-Conde, Eder Alonso Iglesias, José Maria Mostaza Prieto

**Affiliations:** 1 Cardiology Service, Arnau de Vilanova Hospital, Valencia, Spain; 2 Pharmacy Service, Miguel Servet University Hospital, Zaragoza, Spain; 3 Hospital Pharmacy Service, Nuestra Señora de Candelaria University Hospital, Santa Cruz de Tenerife, Spain; 4 Daiichi Sankyo Spain, Madrid, Spain; 5 Lipids and Atherosclerosis Unit, Internal Medicine Service, La Paz-Carlos III Hospital, Madrid, Spain; Università degli Studi di Milano, ITALY

## Abstract

Achieving low-density lipoprotein cholesterol (LDL-C) targets remains challenging for Spanish patients at high (HR) or very-high (VHR) cardiovascular risk, despite treatment with statins and/or ezetimibe (EZE). Escalation to PCSK9 inhibitors (PCSK9i) or inclisiran (INC) is limited due to budgetary concerns. This study evaluates the impact of incorporating bempedoic acid (BA) into lipid-lowering treatment algorithms on LDL-C target attainment and treatment costs. A Monte Carlo simulation used real-world data from 34,967 Spanish adults with HR (29%) or VHR (71%) and uncontrolled LDL-C despite ≥4 weeks of statin treatment with or without EZE. Four treatment sequences were assessed, comparing strategies with and without BA prior to escalating to PCSK9i or INC. In cases where BA did not achieve control, the treatment was switched to PCSK9i/INC, with the BA effect reversed. After simulating the effect of EZE treatment, 17% of patients achieved LDL-C targets. Adding BA enabled an additional 32% of patients to achieve control. Subsequent escalation to injectables further improved control (+49% PCSK9i; + 39% INC). Direct escalation to PCSK9i/INC achieved the same overall control levels (98% PCSK9i, 88% INC) but at higher costs. Incorporating BA before injectables reduced annual treatment costs by almost 30% (−29.8% PCSK9i; −27.2% INC). Introducing BA prior to injectable therapies offers a clinically effective strategy for LDL-C management in HR and VHR patients, significantly reducing budget impact while maintaining high control rates.

## Introduction

Elevated levels of low-density lipoprotein cholesterol (LDL-C) are associated with a higher risk of developing atherosclerotic cardiovascular disease (ASCVD) and related mortality [1,2]. The 2021 European Society of Cardiology (ESC) guidelines recommend lipid-lowering therapies (LLTs) to reduce LDL-C levels, particularly for patients at high cardiovascular disease (CVD) risk [3]. LLTs include statins, ezetimibe (EZE), proprotein convertase subtilisin/kexin type 9 inhibitors (PCSK9i) — evolocumab (E), alirocumab (A), and inclisiran (INC), which can be administered as monotherapy or in combination [3]. The 2021 ESC guidelines recommend adding EZE for patients who do not achieve their CVD risk-based LDL-C target (high-risk patients: < 70 mg/dL; very-high-risk patients: < 55 mg/dL) with the maximum tolerated dose of statins [3]. If patients still do not achieve their LDL-C targets with the combination of statins and EZE, the addition of a PCSK9i is recommended for patients with or without ASCVD who have above-target LDL-C levels [3].

Despite advancements in LLT, achieving LDL-C targets level remains a critical challenge [4–6]. Studies suggest that, in Spain, only 22.0%–23.1%, and 25.0%–27.9% of high- and very high-risk patients, respectively, meet LDL-C targets [5,7,8]. Although being safe and effective, PCSK9i are expensive. Also, their reimbursement in Spain is restricted to at-risk patients with LDL-C > 100 mg/dL, which is well above the thresholds recommended by the 2021 ESC guidelines [3,9,10].

Bempedoic acid (BA), a novel therapy that lowers hepatic cholesterol synthesis, was approved by the European Medicines Agency (EMA) in 2020 for the treatment of primary hypercholesterolemia and mixed dyslipidemia [11,12]. In 2024, approval was extended to include patients with established or at high-risk for developing ASCVD [11,12]. In clinical trials, BA combined with the highest tolerated statin therapy significantly lowered LDL-C levels by 18%–38%, whether used as a monotherapy or in fixed-dose combination (FDC) with EZE respectively, without increasing the rate of adverse events compared to placebo [5,11,13–15]. A study conducted in Germany supports the cost-saving potential of BA treatment [16]. The study suggested that adopting BA for managing LDL-C levels in patients with high or very-high CVD risk would likely lead to significant cost savings, as its annual treatment costs are lower than the cost of PCSK9i [16]. This simulation study used a Monte Carlo approach to estimate that 61.9% of German outpatients at high or very-high CVD risk could achieve LDL-C targets after sequential treatment with BA and EZE, reducing the need for PCSK9i from 66.6% to 37.8% and lowering drug costs by 35.9% annually [16]. In 2024, a consensus document was published in Spain including BA in the therapeutic algorithm for patients with chronic vascular event risk control before escalating to PCSK9i [17,18].

This study aims to evaluate the potential impact of using BA treatment before escalating to PCSK9i or INC on LDL-C targets, drug costs, and prevented ASCVD events in patients with high or very-high CV risk in Spain using real-world patient data and a Monte Carlo simulation approach.

## Methods

### Study design

This non-interventional, longitudinal retrospective study used a Monte Carlo simulation model to analyze secondary anonymized patient-level data, extracted from the IQVIA Electronic Medical Record (EMR) database, covering October 2022 to September 2023. The IQVIA EMR database contains longitudinal anonymized patient real-world data (RWD) since 2013, updated monthly directly from prescriber software. It represents the entire public health care infrastructure of three distinct regions in Spain, comprising approximately 1.2 million patients, representing 3% of the Spanish population. The database includes information on reimbursed retail drugs and outpatient drugs dispensed in hospital pharmacies.

### Study objective

The present study primarily aims to estimate the impact of introducing BA treatment before PCSK9i or INC on achieving LDL-C targets, minimizing the risk of ASCVD events, and reducing drug costs. Specifically, the impact of administering BA in patients who are not controlled even after treatment with statin plus EZE before escalating to PCSK9i (evolocumab/alirocumab [E/A]) or INC is compared to the direct use of PCSK9i or INC without BA.

### Monte Carlo simulation

The Monte Carlo method was used to simulate treatment effects on LDL-C levels for each patient included in the study. In each simulation step, drug efficacy – expressed as the percentage reduction in LDL – was applied, with 10,000 runs conducted per patient. The mean differences in percentage changes in LDL-C levels for LLTs, obtained from a network meta-analysis by Toth et al. (2022) [19], were as follows: EZE, −24.5% (95% CI: −27.5% to −21.5%); BA, −22.8% (95% CI: −26.8% to −18.8%); E/A, −63.7% (95% CI: −67.6% to −57.9%); and INC, −50.2% (95% CI: −55.0% to −45.4%). This network meta-analysis included forty-eight relevant randomized clinical trials (RCTs) and provided comparable efficacy data for all LLTs in our study. Additionally, the NMA included RCTs where over 70% of participants were on moderate-to-high-intensity statins (unless statin-intolerant). Subsequently, for each patient, the mean of the 10,000 new LDL-C values was calculated. Each of the 10,000 runs yields slightly different values due to the confidence intervals (CIs) of the efficacy parameters. The number and proportion of patients achieving LDL-C targets were reported after each treatment simulation.

### Patient selection

Spanish patients aged 18 years and older with high or very-high CVD risk, as defined by the 2021 ESC guidelines, were included in the study cohort if they had been on LLTs - necessarily including a statin – for at least 4 weeks during the study period and had available LDL-C results to enable simulation of treatment effects. Patients who were already receiving BA or PCSK9i, or who had already achieved LDL-C targets, were excluded from the simulation cohort. The simulation focused on individuals treated with statins alone or in combination with EZE, whose LDL-C remained above target thresholds. These patients were assumed to be on their maximum tolerated statin dose or not receiving statins due to documented intolerance. Consequently, no simulation was performed to add or intensify statin therapy. Compliance with current therapy was not directly measured in the EMR data and was assumed to be optimal for the purposes of simulation. According to the 2021 ESC guidelines, the LDL-C target level is < 55 mg/dL (<1.4 mmol/L) for very-high-risk patients and <70 mg/dL (<1.8 mmol/L) for high-risk patients.

### Patient classification

The index date was defined as the date of the last LDL-C measurement with at least 4 weeks on stable LLT. Patients were classified as high or very-high CVD risk based on the 2021 ESC guideline, along with Systematic Coronary Risk Evaluation 2 (SCORE2) and SCORE2-Older Persons (OP) criteria [3].

### Simulation drug algorithm

**Fig 1:**
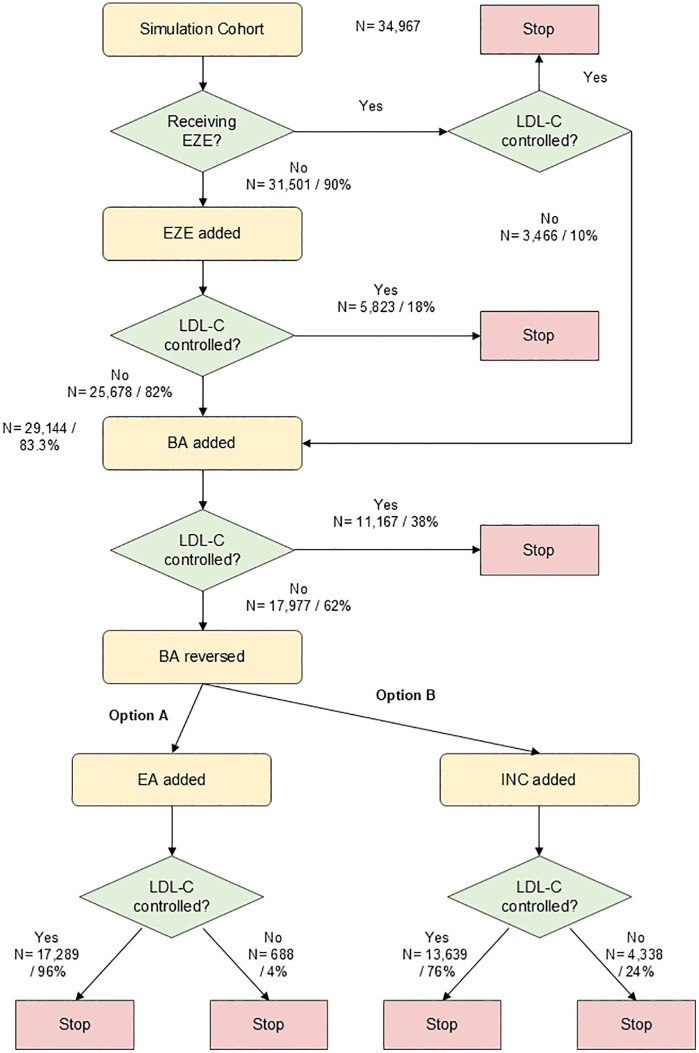
Monte Carlo simulation algorithms for evaluating the effects of sequential treatment with EZE, BA, and PCSK9i or INC. BA, bempedoic acid; E/A, evolocumab/alirocumab; EZE, ezetimibe; INC, inclisiran; LDL-C, low-density lipoprotein cholesterol; PCSK9i, proprotein convertase subtilisin/kexin type 9 inhibitors. LDL-C controlled: Patients whose LDL-C levels reach the target level as per 2021 ESC guidelines are considered LDL-C controlled.

**Fig 2:**
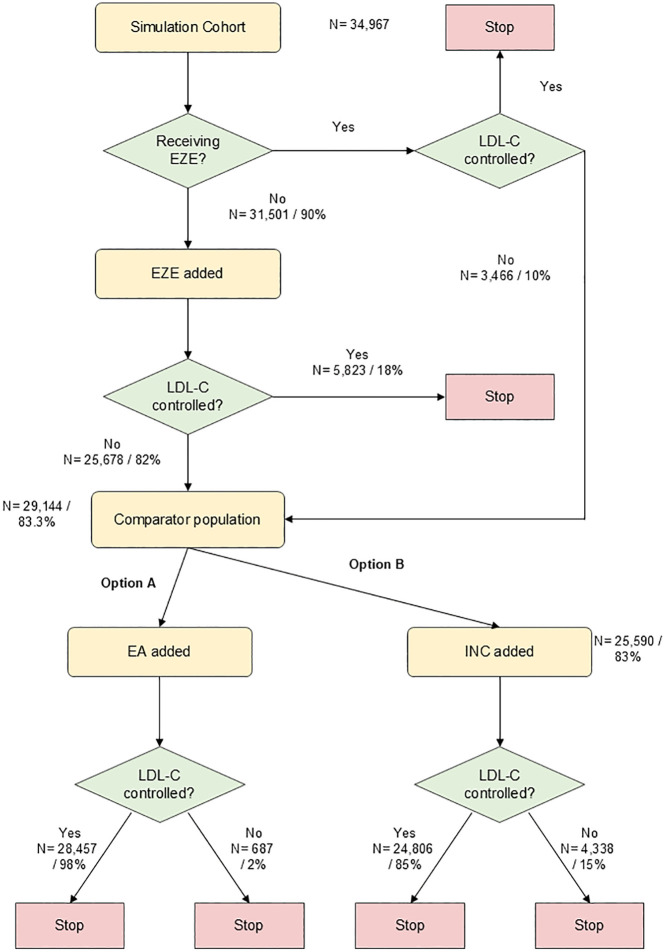
Monte Carlo simulation algorithms for evaluating the treatment effects without BA (comparator scenario). E/A, evolocumab/alirocumab; EZE, ezetimibe; INC, inclisiran; LDL-C, low-density lipoprotein cholesterol; PCSK9i, proprotein convertase subtilisin/kexin type 9 inhibitors. LDL-C controlled: Patients whose LDL-C levels reach the target level as per 2021 ESC guidelines are considered LDL-C controlled.

The simulation initially assessed the impact of EZE treatment for patients who did not receive this drug and did not achieve LDL-C targets. Subsequently, BA was introduced for patients who remained uncontrolled despite EZE therapy. In cases where LDL-C targets were still not met after BA treatment simulation, the effect of BA was reversed, and the model simulated the treatment effects of PCSK9i (E/A) or INC (Fig 1). The model assumed an immediate transition to the next therapy if the response to treatment was inadequate. When a therapy switch occurred, the cost of BA treatment and its LDL-C reduction effect were excluded, and the simulation proceeded based on the mean LDL-C levels post-EZE treatment. This approach was compared using a comparator scenario that used the same population as the BA simulation (Fig 2). The comparator scenarios included simulations of PCSK9i or INC effects on patients receiving statins who did not achieve LDL-C targets after EZE, without BA addition.

### Budget impact

Our model assumed instantaneous treatment outcomes and therapy switch when LDL-C control was not achieved. As a result, the cost of treatment was only applied for the final treatment, i.e., when the patient achieved LDL-C control or had exhausted treatment options. As all patients were on statin treatment, this cost was not included in the model. Official prices were considered to compute the annual treatment costs for each therapy. The annual treatment cost for EZE was estimated at €402 from the retail price (including value-added tax) published by the Ministry of Health [20]. For BA, the annual cost was €1,020, based on a daily pill and the retail price of €78. The annual cost for E/A was €6,278, based on an injection every 2 weeks and the retail price of €482 [20]. Based on administration differences and the retail price of €2,348, the cost of INC was €7,045 in the first year and €4,696 in subsequent years [20]. Only the subsequent years’ cost was utilized in this study for simplifying the methodology. For budget impact calculations, retail prices were used, as indicated in the latest pharmacoeconomic evaluation guidelines by CPAF and GENESIS [20]. Annual costs of E/A and INC were estimated according to molecule type and pack size. Costs were calculated as drug consumption in mg multiplied by the cost per mg for each molecule and dose, using the retail price in Spain for the packs with the lowest cost per mg. All costs reflect official retail prices and represent a payer perspective based on list prices. Real-world NHS acquisition costs may differ due to negotiated discounts or risk-sharing agreements, and the present estimates should be interpreted accordingly. For INC, only the maintenance-year cost (€4,696) was applied in the base-case analysis; a sensitivity analysis using the first-year cost (€7,045) is reported in Supporting Information (S4 Table).

### Prevented major adverse cardiovascular events

The prevented 4-point major adverse cardiovascular events (4P-MACE, a composite of coronary heart death, myocardial infarction, ischemic stroke, or coronary revascularization) was estimated based on LDL-C reduction results from the BA simulation scenarios compared to the comparator scenarios without BA. The baseline 4P-MACE rate was defined as the first occurrence of any 4P-MACE within 12 months of the index. A baseline annual 4P-MACE incidence rate of 3.0% was assumed for the primary analysis, agreed with the clinical expert co-authors as a conservative and clinically appropriate estimate for this cohort (71% VHR, 39.2% ASCVD, 37.1% DM), consistent with published real-world data in comparable populations [21]. The sensitivity of results to this assumption is explored across rates of 2.0%–4.0% in Supporting Information (S3 Table). The annual prevented events per patient in scenarios with and without BA were estimated, and the absolute and relative differences in prevented events were compared. Based on the 2015 CTTC meta-analysis, a 1 mmol/L (38.67 mg/dL) reduction in LDL-C was considered equivalent to a 21% relative risk reduction (RRR) of total 4P-MACE [22,23].

For each individual patient, the absolute risk reduction (ARR) in 4P-MACE was derived by applying the RRR, calculated from that patient’s simulated LDL-C reduction at each treatment step using the CTTC 21% per mmol/L estimate, to the baseline annual 4P-MACE event probability (assuming a maximum of one event per patient within 12 months of the index date). The resulting ARR represents the individual number of events prevented per patient, and cohort-level results were obtained by aggregating these individual estimates. The RRR was applied sequentially at each simulation step, calculated from each individual patient’s LDL-C level at the start of that treatment step rather than from the untreated baseline, consistent with Katzmann et al. (2022) [16]. Prevented MACE events were estimated across three sequential steps, each applied at the individual patient level: (1) EZE step (shared identically between both arms), the RRR was applied to the LDL-C reduction achieved by EZE for each individual patient. For patients not yet on EZE (n = 31,501), this reflects the reduction from each patient’s baseline LDL-C to their post-EZE simulated level; for patients already on EZE (n = 3,466), no additional LDL-C reduction was attributed. Prevented events at this step are identical across both arms. (2) BA step (BA-inclusive arm only), for each patient achieving LDL-C control with BA (n = 11,167; 38.3% of BA-eligible patients), the RRR was applied to that patient’s individual LDL-C reduction from their post-EZE level to their post-BA simulated level. (3) E/A or INC step, for each patient not achieving control at BA (n = 17,977), the BA effect was reversed to restore that patient’s post-EZE LDL-C level, and the RRR was applied to their individual LDL-C reduction from that post-EZE level following escalation to E/A or INC. In the comparator arm (no BA), the RRR was applied to each patient’s individual LDL-C reduction from their post-EZE level upon escalation to E/A or INC (n = 29,144). This approach ensures that the injectable step starts from an equivalent LDL-C level in both arms for non-BA-controlled patients, and that the differential in prevented events between arms arises solely from the additional LDL-C reduction achieved by BA in patients who respond to it.

All statistical analyses were conducted using Statistical Analysis System (SAS) software version 9.2 (SAS Institute Inc., Cary, NC).

## Results

### Study and simulation cohort

**Fig 3:**
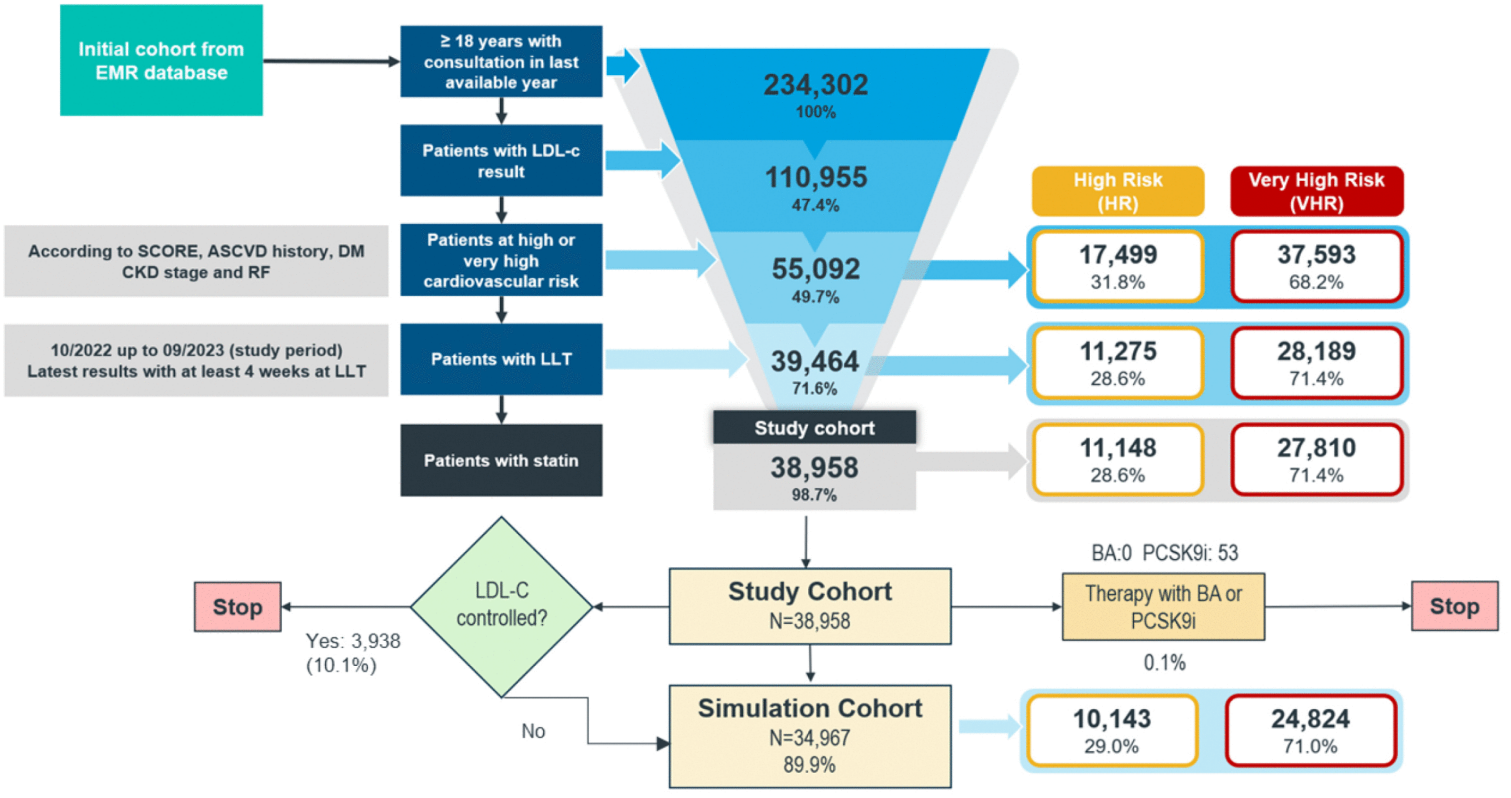
Study cohort and simulation cohort sequential flowchart. ASCVD: Atherosclerotic cardiovascular disease; BA: Bempedoic acid; CKD: Chronic kidney disease; DM: Diabetes Mellitus; EMR: IQVIA Electronic Medical Record (EMR) database; LDL-C: Low density lipoprotein cholesterol. LLT: Lipid lowering therapy; PCSK9i: Proprotein convertase subtilisin/kexin type 9 inhibitors. LDL-C controlled: Patients whose LDL-C levels reach the target level as per 2021 ESC guidelines are considered LDL-C controlled.

In the EMR database, a total of 55,092 patients aged 18 years or older at high or very-high CVD risk (high-risk: 17,499 [32%], very high-risk: 37,593 [68%]) whose LDL-C results were accessible and who had at least one consultation between October 2022 and September 2023 were identified from a population database of around 1.2 million patients in Spain. Of these patients, 39,464 (71.6%) were on LLT for at least 4 weeks during the study period. Of these patients, 506 were excluded, as they were not receiving statin treatment, and 53 more for already being on PCSK9i therapy. Additionally, 3,938 were excluded, as they had already achieved LDL-C targets. Finally, 34,967 patients (high-risk: 10,143 [29%]; very high-risk: 24,824 [71%]) were included in the simulation cohort (Fig 3).

The baseline characteristics of the simulation cohort are presented in Table 1. The average age was 70.7 ± 10.8 years, with a high percentage of females (50.9%). Patients with very high-risk had a higher average age compared to those with high-risk (74.4 vs. 61.8 years). The average LDL-C level was 109.1 ± 39.8 mg/dL, with higher levels observed in high-risk patients compared to the very-high-risk patients (133.6 vs. 99.1 mg/dL). Hypertension was the most common risk factor (66.5%), followed by diabetes mellitus (DM) (37.1%), moderate chronic kidney disease (CKD) (2.8%), and severe CKD (0.5%), with 4.1% of patients being current smokers and 14.8% having a previous smoking history. Additionally, 39.2% of patients were diagnosed with ASCVD. Most patients received moderate-intensity statins (65.9%), followed by high-intensity (22.1%) and low-intensity (12.0%) statins prior to inclusion in the simulation cohort.

**Table 1:** Baseline characteristics of the simulation cohort.

Variables	High risk (N = 10,143)	Very high risk (N = 24,824)	Simulation Cohort (N = 34,967)
Sex, female, n (%)	5,483 (54.1%)	12,320 (49.6%)	17,803 (50.9%)
Age (years), mean (SD)	61.8 (8.5)	74.4 (9.4)	70.7 (10.8)
LDL-C (mg/dL), mean (SD)	133.6 (46.1)	99.1 (31.8)	109.1 (39.8)
DM, n (%)	2,504 (24.7%)	10,458 (42.1%)	12,962 (37.1%)
Hypertension, n (%)	5,402 (53.3%)	17,866 (72.0%)	23,268 (66.5%)
Moderate CKD, n (%)	331 (3.3%)	637 (2.6%)	968 (2.8%)
Severe CKD, n (%)	0 (0.0%)	178 (0.7%)	178 (0.5%)
ASCVD, n (%)	0 (0.0%)	13,710 (55.2%)	13,710 (39.2%)
Smoker, n (%)	565 (5.6%)	884 (3.6%)	1,449 (4.1%)
Ex-smoker, n (%)	1,964 (19.4%)	3,213 (12.9%)	5,177 (14.8%)
Statin intensity of last LLT, n (%)			
High	1348 (4.1%)	6397 (18.6%)	7745 (22.1%)
Low	1148 (3.3%)	3037 (8.4%)	4185 (12.0%)
Moderate	7647 (22.7%)	15390 (43.0%)	23037 (65.9%)

CKD, chronic kidney disease; DM, diabetes mellitus; LLT, lipid-lowering therapy; SD, standard deviation.

### Drug simulation algorithm results: Controlled and non-controlled patients

**Fig 4:**
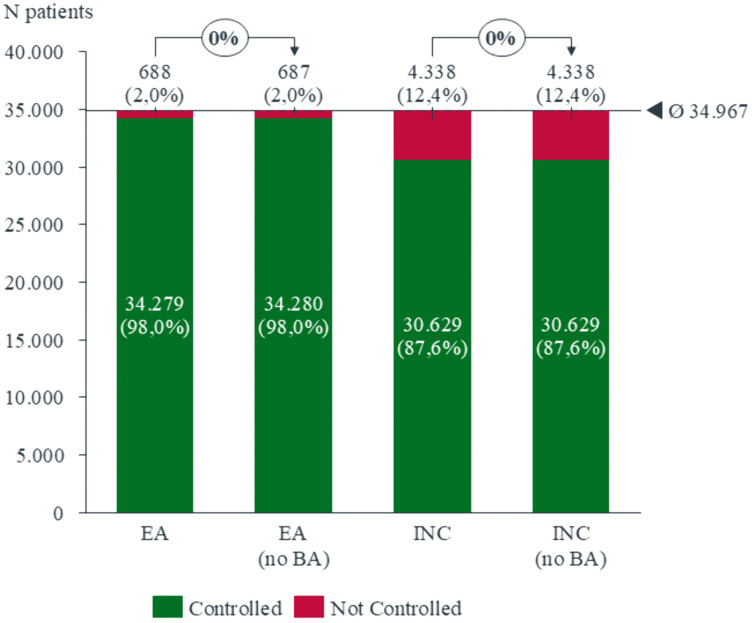
LDL-C level controlled versus non-controlled population simulation results. BA, bempedoic acid; E/A, evolocumab/alirocumab; INC, inclisiran. LDL-C controlled: Patients whose LDL-C levels reach the target level as per 2021 ESC guidelines are considered LDL-C controlled.

The simulation results show that the introduction of BA before E/A or INC therapy did not affect the final number of patients achieving LDL-C targets (Fig 4). This is because patients who failed to meet LDL-C targets with BA were subsequently switched to E/A or INC. Thus, approximately 98.0% (n = 34,279; high-risk: 9,862, very high-risk: 24,417) of patients achieved LDL-C targets after the introduction of a sequential add-on treatment of EZE, BA and, in case of not achieving the goal, BA withdrawal and E/A addition, and the same proportion of patients (98.0%, n = 34,280; high-risk: 9,863, very high-risk: 24,417) achieved LDL-C targets with EZE, directly followed by E/A therapy. When the sequential add-on treatment with BA was introduced before INC therapy, 87.6% (n = 30,629; high-risk: 8,198, very high-risk: 22,431) of patients achieved LDL-C targets, identical to the 87.6% (n = 30,629; high-risk: 8,198, very high-risk: 22,431) achieving LDL-C targets with EZE followed by INC therapy. While only 16.7% of the population achieved LDL-C targets with statins plus EZE, the addition of BA enabled 48.6% of patients to achieve the LDL-C goal before starting E/A or INC (See supporting information, S1 Fig). Therefore, treatment with BA reduced the need for E/A and INC therapy by 31.9%.

### Budget impact

**Fig 5:**
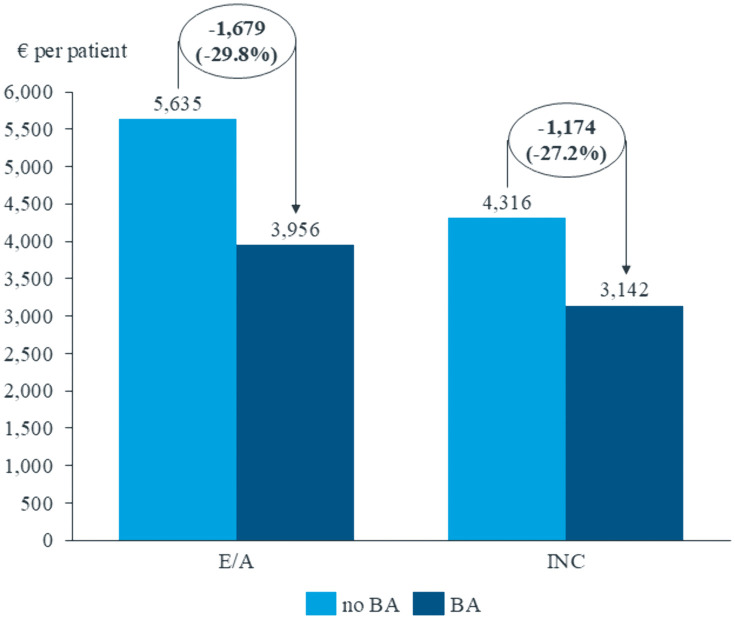
Annual treatment cost per patient. BA, bempedoic acid; E/A, evolocumab/alirocumab; INC, inclisiran.

Introducing BA before E/A or INC therapy resulted in lower annual treatment costs per patient (Fig 5). Specifically, the introduction of a sequential add-on treatment with BA before E/A therapy translated to an annual treatment cost per patient of €-1,679, 29.8% lower than without BA, whereas introducing this same treatment before INC therapy resulted in a 27.2% (€-1,174) lower treatment cost per patient. This annual treatment cost reduction was due to the difference in the annual treatment cost of BA (€1,020) and E/A (€6,278) or INC (€4,696), as patients who achieved LDL-C target levels with BA did not need further treatment with E/A nor INC.

### LDL-C absolute reduction and prevented 4P-MACE

**Fig 6:**
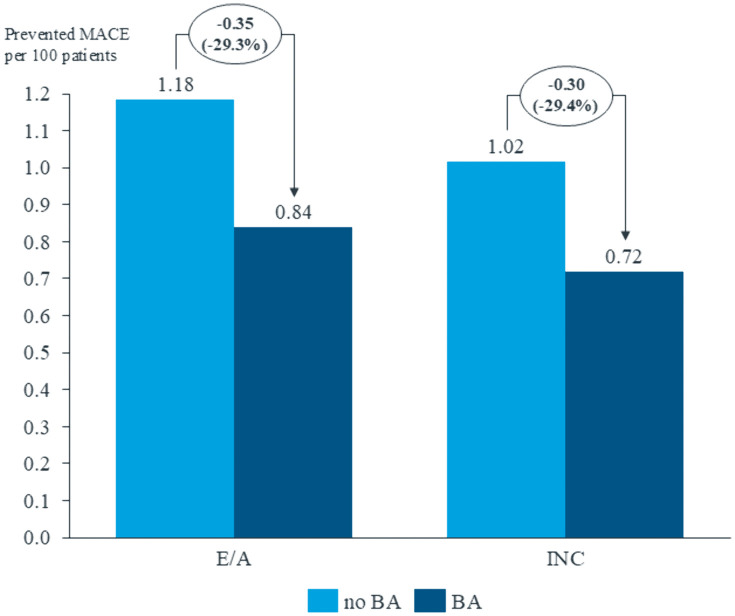
Number of 4PMACE per 100 patients prevented annually. BA, bempedoic acid; MACE, major adverse cardiovascular events. E/A, evolocumab/alirocumab; INC, inclisiran.

Absolute LDL-C reduction was higher in the scenario without BA than with BA. Consequently, sequential add-on treatment with EZE and BA before E/A therapy was estimated to prevent 0.35 fewer events per 100 patients per year of 4P-MACE compared to the EZE followed by E/A regimen, whereas introducing BA before INC therapy prevented 0.30 fewer events per 100 patients per year compared to the EZE followed by INC regimen (Fig 6; sensitivity analyses across baseline rates of 2.0%–4.0% are presented in S3 Table).

## Discussion

RWD from the Spanish EMR database from October 2022 to September 2023 identified 38,905 patients at high or very-high CVD risk, with LDL-C results available, receiving only a statin or a statin plus EZE, of whom 89.9% were not achieving the LDL-C targets recommended by the 2021 ESC guidelines. In this study, we simulated real-world patient data to assess the efficacy of further LDL-C reduction using alternative treatment strategies. These strategies included directly adding PCSK9i or INC or incorporating BA before escalating to PCSK9i or INC. Then we estimated the proportion of patients achieving LDL-C targets, the treatment costs, and the number of MACE prevented based on LDL-C absolute reductions.

The simulation results show that 48.6% of patients achieved LDL-C targets after treatment with BA, while the remaining 51.4% needed PCSK9i therapy. Incorporating BA into the treatment algorithm allowed 31.9% of patients to avoid PCSK9i treatment. It also resulted in savings of €1,679–€1,174 (E/A-INC) per patient without affecting the proportion of patients reaching LDL-C targets. Overall, 98.0% of patients achieved LDL-C targets with sequential therapy using EZE, BA, and E/A, while 87.6% achieved targets with EZE, BA, and INC. The benefits of incorporating BA have been tested through Monte Carlo simulations in other countries, such as USA, Austria, and Germany, and are consistent with those from our study [14,24,25]. The results of the E/A arm are comparable to a previous German study using a similar Monte Carlo simulation algorithm, where sequential add-on therapy with EZE, BA, and a PCSK9i (or EZE followed by PCSK9i) successfully controlled LDL-C levels in 97.7% of high or very-high CVD risk patients [16].

All simulations in our study were performed in accordance with consensus documents, ensuring alignment with their recommendations [17,26–28]. Our study supports the recent consensus of Spanish scientific societies (*Sociedad Española de Cardiología* [SEC] and Sociedad Española de Arteriosclerosis [SEA]), recommending BA introduction in patients with high or very-high CVD risk before starting PCSK9is treatment, as this strategy could lead to substantial savings for the Spanish National Health Service (NHS) without compromising clinical control [17,27,29].

Clinical trials have shown that PCSK9i can effectively lower LDL-C levels, potentially leading to a reduction in the risk of 4PMACEs [19]. Our simulations suggest that direct escalation to PCSK9i may lead to approximately 0.4 fewer events per 100 patients per year compared to a sequential therapy approach involving BA, primarily due to the higher absolute LDL-C reduction achieved by PCSK9i. In both treatment strategies, most patients reach their LDL-C targets based on their cardiovascular risk. Therefore, the added benefit of further lowering LDL-C levels beyond these goals comes at an added cost to the health care system. This is particularly relevant in Spain, where reimbursement restrictions on PCSK9i limit patient eligibility for treatment in clinical practice. It is important to note that our study did not account for these reimbursement constraints and thus underestimates the potential clinical benefits of BA for Spanish patients. In the FOURIER and ODYSSEY OUTCOMES studies, E/A prevented 15% more 4PMACEs than placebo. BA prevented 13% more in the CLEAR OUTCOMES study [30–32].

In our study, about 85% of the patient cohort were on moderate-to-high-intensity statins, similar to RCT populations in the NMA used for efficacy parameters. Furthermore, this NMA provides the mean differences in LDL-C levels as percentage changes with 95% CIs, which are robust for use in Monte Carlo simulations. The NMA also explicitly explained the exclusion of certain previously published RCTs. For these reasons, this NMA has also been used in other simulation studies as well [33,34].

Furthermore, it is important to acknowledge that the IQVIA EMR database is based on RWD inherently reflecting the clinical practices of health care professionals. Consequently, missing data is common and must be considered. As LDL-C values were essential for the efficacy simulation, LDL-C availability was an inclusion criterion for the cohort. Therefore, certain baseline characteristics, such as smoking status, may differ from those reported in similar studies. For instance, applying the LDL-C availability criterion resulted in the loss of data on smokers, reducing their frequency to 4.1%, compared to 16.1% in the SANTORINI study.

A clinically relevant subgroup for future investigation is patients with DM, who comprised 37.1% of our simulation cohort and are overrepresented in the very-high-risk stratum (42.1%). Evidence suggests that bempedoic acid provides significant and consistent LDL-C reduction and MACE-4 risk reduction across glycemic strata, with potentially greater absolute benefit in patients with DM given their higher baseline cardiovascular risk, and without increasing HbA1c levels or new-onset diabetes risk [35,36]. Future simulation studies should consider stratified analyses by diabetes status to quantify the differential impact on LDL-C management and cost reduction in this high-risk subgroup.

### Limitations

This study has several limitations that should be considered. The analysis included only direct drug costs, consistent with previous simulation studies [14,25], while administration costs and indirect savings were not accounted for, which may underestimate the true economic impact. In a similar study, Blaum et al. (2021) projected that incorporating BA before PCSK9i would still reduce treatment costs even when accounting for prevented-event savings [37]. Cost estimates are based on official list prices published by the Spanish Ministry of Health, as recommended by the CAPF/GENESIS pharmacoeconomic evaluation guidelines. In practice, the Spanish NHS may acquire drugs at negotiated prices below list, and BA, E/A, and INC are all reimbursed under restricted indications and special funding conditions, such as expenditure ceilings, which affect the final cost to the NHS. Furthermore, the simulation did not account for current Spanish reimbursement criteria for PCSK9i, which restrict eligibility to patients with LDL-C > 100 mg/dL, well above ESC guideline targets, meaning the benefit of BA would likely be greater if these thresholds were applied, as BA would represent the only escalation option for many patients after statin and EZE. The real cost savings of BA addition before INC may also be higher than estimated, as only the maintenance-year cost of INC (€4,696) was applied in the base case rather than the higher initiation cost (€7,045); a sensitivity analysis using the first-year cost is reported in S4 Table.

The model assumed immediate treatment effects, instantaneous therapy switching, and no discontinuation due to adverse events or non-adherence, which may not reflect clinical practice. Drug costs were applied only for the final treatment step, that is, when the patient achieved LDL-C control or exhausted available options. Drug costs were applied only for the final treatment step, and intermediate costs during therapy switching were not modelled, which may underestimate total costs. The simulation model assumes optimal adherence and does not account for real-world discontinuation rates, titration delays, or differences in persistence across therapies. In clinical practice, oral therapies such as BA may demonstrate higher long-term adherence compared with injectable regimens due to the convenience of once-daily oral administration, while bi-annual injectable regimens (INC) may benefit from higher persistence once initiated. These adherence differences could meaningfully affect long-term cost and clinical outcomes [38–40].

Efficacy parameters from the Toth et al. (2022) NMA were applied uniformly across the simulation cohort, without stratification by baseline LDL-C level, statin intensity, or cardiovascular risk category, which may not fully capture the heterogeneity of LDL-C response observed in clinical practice [19]. The IQVIA EMR database covers three health regions in Spain, representing approximately 3% of the national population (~1.2 million patients). While prior studies have confirmed its demographic representativeness of the broader Spanish population, generalizability to all Spanish regions should be interpreted with appropriate caution.

The estimated difference in prevented 4P-MACE events between BA-inclusive and BA-free strategies (0.2–0.5 events per 100 patients per year across the range of plausible baseline incidence rates tested in sensitivity analyses) is modest and sensitive to the assumed baseline annual event rate and the CTTC-derived RRR per mmol/L LDL-C reduction. Furthermore, the present model is limited to a 1-year time horizon. Long-term modelling incorporating time-discounting, treatment discontinuation, and event-free survival beyond 12 months would provide a more complete health-economic picture and is recommended for future work. Finally, subgroup analyses by diabetes status, sex, or statin intensity were not performed. Given the high prevalence of DM in this cohort (37.1%) and emerging evidence of consistent or greater BA benefit in this subgroup, stratified analyses are recommended for future work.

## Conclusions

The results of this study suggest that sequential treatment with BA followed by PCSK9i, or INC would enable the same percentage of high- and very-high-risk Spanish patients to achieve LDL-C target levels as without BA, although fewer 4P-MACE would be prevented. Furthermore, introducing BA before PCSK9i could reduce NHS treatment costs by a third. Our study shows that following Spanish scientific societies’ recommendation to introduce BA before starting PCSK9i or INC treatment in patients with high or very-high CVD risk would lead to substantial savings for the Spanish NHS without compromising clinical control.

## Supporting information

S1 Fig Dispersion of individual LDL-C level values for high- and very-high-risk patients after each drug simulation effect following the therapeutic algorithm. BA, bempedoic acid; E/A, evolocumab/alirocumab; EZE, ezetimibe; INC, inclisiran; LDL-C, low-density lipoprotein cholesterol; HR: high-risk; VHR: very-high risk. Color code: Red, population with LDL-C not controlled; Green, population with LDL-C controlled; Gray, initial simulation cohort.
(TIF)

S2 Fig Patient treatment flow for high-risk patients. BA, bempedoic acid; E/A, evolocumab/alirocumab; EZE, ezetimibe; INC, inclisiran; LDL-C, low-density lipoprotein cholesterol; mLDL, mean LDL-C; HR: high-risk; VHR: very-high risk.
(TIF)

S3 Fig Patient treatment flow for very-high-risk patients. BA, bempedoic acid; E/A, evolocumab/alirocumab; EZE, ezetimibe; INC, inclisiran; LDL-C, low-density lipoprotein cholesterol; mLDL, mean LDL-C; HR: high-risk; VHR: very-high risk.
(TIF)

S4 Fig Patient treatment flow for high- and very-high-risk patients. BA, bempedoic acid; E/A, evolocumab/alirocumab; EZE, ezetimibe; INC, inclisiran; LDL-C, low-density lipoprotein cholesterol; mLDL, mean LDL-C; HR: high-risk; VHR: very-high risk.
(TIF)

S1 Table Study cohort flow. BA, bempedoic acid; E/A, evolocumab/alirocumab; EZE, ezetimibe; INC, inclisiran; LDL-C, low-density lipoprotein cholesterol; HR: high-risk; VHR: very-high risk.
(DOCX)

S2 Table Mean LDL at each treatment step. BA, bempedoic acid; E/A, evolocumab/alirocumab; EZE, ezetimibe; INC, inclisiran; LDL-C, low-density lipoprotein cholesterol; HR: high-risk; VHR: very-high risk.
(DOCX)

S3 Table Linear sensitivity analyses for prevented 4P-MACE under alternative baseline annual event rates. Total MACE events prevented per 100 patients, by simulation step, arm, and baseline 4P-MACE annual incidence rate (2.0%–4.0%). BA, bempedoic acid; MACE, major adverse cardiovascular events; E/A, evolocumab/alirocumab; INC, inclisiran.
(DOCX)

S4 Table Sensitivity analysis for annual costs using inclisiran first-year versus maintenance-year cost assumptions. BA, bempedoic acid; INC, inclisiran. Note: Cost per patient values are weighted averages across the full simulation cohort (N = 34,967), accounting for the proportion of patients controlled at each treatment step (EZE, BA, or INC). Values are not limited to patients escalated to INC.
(DOCX)
