# Supplementary material for: LDL-C target attainment and treatment costs in high-risk and very-high-risk patients with or without bempedoic acid: A Spanish cohort simulation
Source: PLoS One. 2026 Aug 14;21(8):e0353193. doi: 10.1371/journal.pone.0353193 (PMC13475820; doi:10.1371/journal.pone.0353193)
Supplement: Supplementary file 1 [file pone.0353193.s005.docx]

**S5 Table. Study cohort flow**

| **Patients study cohort** | **HR (N)** | **VHR (N)** | **Total (N)** |
| --- | --- | --- | --- |
| Total not controlled patients | 10,143 | 24,824 | 34,967 |
| Already on EZE (and not controlled) | 847 | 2,619 | 3,466 |
| Entered EZE simulation | 9,296 | 22,205 | 31,501 |
| Controlled at EZE | 1,725 | 4,098 | 5,823 |
| Not controlled at EZE | 7,571 | 18,107 | 25,678 |
| Entered BA step | 8,418 | 20,726 | 29,144 |
| Controlled at BA | 3,084 | 8,083 | 11,167 |
| Not controlled at BA | 5,334 | 12,643 | 17,977 |
| Entered E/A or INC | 5,334 | 12,643 | 17,977 |
| E/A Intervention arm: Controlled | 5,053 | 12,236 | 17,289 |
| E/A Intervention arm: Not controlled | 281 | 407 | 688 |
| E/A Comparator arm (no BA): Controlled | 8,138 | 20,319 | 28,457 |
| E/A Comparator arm (no BA): Not controlled | 280 | 407 | 687 |
| INC Intervention arm: Controlled | 3,389 | 10,250 | 13,639 |
| INC Intervention arm: Not controlled | 1,945 | 2,393 | 4,338 |
| INC Comparator arm (no BA): Controlled | 6,473 | 18,333 | 24,806 |
| INC Comparator arm (no BA): Not controlled | 1,945 | 2,393 | 4,338 |

BA, bempedoic acid; E/A, evolocumab/alirocumab; EZE, ezetimibe; INC, inclisiran; LDL-C, low-density lipoprotein cholesterol; HR: high-risk; VHR: very-high risk.
