# Supplementary material for: LDL-C target attainment and treatment costs in high-risk and very-high-risk patients with or without bempedoic acid: A Spanish cohort simulation
Source: PLoS One. 2026 Aug 14;21(8):e0353193. doi: 10.1371/journal.pone.0353193 (PMC13475820; doi:10.1371/journal.pone.0353193)
Supplement: Supplementary file 2 [file pone.0353193.s006.docx]

**S6 Table. Mean LDL at each treatment step**

| **CV Risk** | **Simulation step** | **Mean LDL (mg/dL)** | **SD** | **N** |
| --- | --- | --- | --- | --- |
| HR | Baseline LDL, all patients | 133.6 | 46.1 | 10,143 |
| HR | Post-EZE simulation | 100.3 | 34.0 | 9,296 |
| HR | Effective post-EZE, all EZE patients | 103.8 | 38.1 | 10,143 |
| HR | Post-BA, all BA patients | 86.6 | 28.0 | 8,418 |
| HR | Post-BA, controlled at BA | 61.9 | 4.6 | 3,084 |
| HR | Post-BA, not controlled (before reverting BA effect) | 101.0 | 25.9 | 5,334 |
| HR | Post-BAE/A, Intervention arm E/A | 47.5 | 12.2 | 5,334 |
| HR | Post-E/A, Comparator arm (no BA) E/A | 40.8 | 13.2 | 8,418 |
| HR | Post-BAINC, Intervention arm INC | 65.2 | 16.7 | 5,334 |
| HR | Post-INC, Comparator arm (no BA) INC | 55.9 | 18.1 | 8,418 |
| VHR | Baseline LDL, all patients | 99.1 | 31.8 | 24,824 |
| VHR | Post-EZE simulation | 75.7 | 23.6 | 22,205 |
| VHR | Effective post-EZE, all EZE patients | 77.1 | 25.3 | 24,824 |
| VHR | Post-BA, all BA patients | 63.8 | 18.5 | 20,726 |
| VHR | Post-BA, controlled at BA | 48.7 | 3.7 | 8,083 |
| VHR | Post-BA, not controlled (before reverting BA effect) | 73.5 | 17.7 | 12,643 |
| VHR | Post-BAE/A, Intervention arm E/A | 34.6 | 8.3 | 12,643 |
| VHR | Post-E/A, Comparator arm (no BA) E/A | 30.0 | 8.7 | 20,726 |
| VHR | Post-BAINC, Intervention arm INC | 47.4 | 11.4 | 12,643 |
| VHR | Post-INC, Comparator arm (no BA) INC | 41.2 | 12.0 | 20,726 |
| Total | Baseline LDL, all patients | 109.1 | 39.8 | 34,967 |
| Total | Post-EZE simulation | 83.0 | 29.3 | 31,501 |
| Total | Effective post-EZE, all EZE patients | 84.8 | 32.0 | 34,967 |
| Total | Post-BA, all BA patients | 70.4 | 24.1 | 29,144 |
| Total | Post-BA, controlled at BA | 52.3 | 7.1 | 11,167 |
| Total | Post-BA, not controlled (before reverting BA effect) | 81.6 | 24.0 | 17,977 |
| Total | Post-BAE/A, Intervention arm E/A | 38.4 | 11.3 | 17,977 |
| Total | Post-E/A, Comparator arm (no BA) E/A | 33.1 | 11.3 | 29,144 |
| Total | Post-BAINC, Intervention arm INC | 52.7 | 15.5 | 17,977 |
| Total | Post-INC, Comparator arm (no BA) INC | 45.4 | 15.5 | 29,144 |

BA, bempedoic acid; E/A, evolocumab/alirocumab; EZE, ezetimibe; INC, inclisiran; LDL-C, low-density lipoprotein cholesterol; HR: high-risk; VHR: very-high risk.
