# Supplementary material for: LDL-C target attainment and treatment costs in high-risk and very-high-risk patients with or without bempedoic acid: A Spanish cohort simulation
Source: PLoS One. 2026 Aug 14;21(8):e0353193. doi: 10.1371/journal.pone.0353193 (PMC13475820; doi:10.1371/journal.pone.0353193)
Supplement: Supplementary file 3 [file pone.0353193.s007.docx]

**S7 Table. Linear sensitivity analyses for prevented 4P-MACE under alternative baseline annual event rates**

| **Total Baseline 4P-MACE annual event rates** | **2.0%** | **2.5%** | **3.0%** | **3.5%** | **4.0%** |
| --- | --- | --- | --- | --- | --- |
|  | **Total MACE events prevented per 100 patients** | | | | |
| E/A Intervention arm | 0.56 | 0.7 | 0.84 | 0.98 | 1.12 |
| E/A Comparator arm (no BA) | 0.79 | 0.99 | 1.18 | 1.38 | 1.58 |
| Difference when adding BA before E/A | −0.23 | −0.29 | −0.35 | −0.40 | −0.46 |
| INC Intervention arm | 0.48 | 0.6 | 0.72 | 0.84 | 0.96 |
| INC Comparator arm (no BA) | 0.68 | 0.85 | 1.02 | 1.19 | 1.36 |
| Difference when adding BA before INC | −0.20 | −0.25 | −0.30 | −0.35 | −0.40 |

Total MACE events prevented per 100 patients, by simulation step, arm, and baseline 4P-MACE annual incidence rate (2.0%–4.0%). BA, bempedoic acid; MACE, major adverse cardiovascular events; E/A, evolocumab/alirocumab; INC, inclisiran.
