# Supplementary material for: LDL-C target attainment and treatment costs in high-risk and very-high-risk patients with or without bempedoic acid: A Spanish cohort simulation
Source: PLoS One. 2026 Aug 14;21(8):e0353193. doi: 10.1371/journal.pone.0353193 (PMC13475820; doi:10.1371/journal.pone.0353193)
Supplement: Supplementary file 4 [file pone.0353193.s008.docx]

**S8 Table. Sensitivity analysis for annual costs using inclisiran first-year versus maintenance-year cost assumptions**

|  | INC maintenance-year cost (€4,696/ patient) | INC first-year cost (€7,045/ patient) |
| --- | --- | --- |
| INC Intervention arm - cost per patient | €3,142 | €4,350 |
| INC Comparator arm (no BA) - cost per patient | €4,316 | €6,274 |
| Difference when adding BA before INC - cost per patient | €1,174 | €1,924 |
| Relative reduction in cost per patient when adding BA before INC | 27.2% | 30.7% |

BA, bempedoic acid; INC, inclisiran.

Note: Cost per patient values are weighted averages across the full simulation cohort (N=34,967), accounting for the proportion of patients controlled at each treatment step (EZE, BA, or INC). Values are not limited to patients escalated to INC.
