# Supplementary material for: LDL-C target attainment and treatment costs in high-risk and very-high-risk patients with or without bempedoic acid: A Spanish cohort simulation
Source: PLoS One. 2026 Aug 14;21(8):e0353193. doi: 10.1371/journal.pone.0353193 (PMC13475820; doi:10.1371/journal.pone.0353193)
Supplement: Supplementary file 5 [file pone.0353193.s010.docx]

# Response to reviewers

**Manuscript title:** LDL-C target attainment and treatment costs in high-risk and very-high-risk patients with or without bempedoic acid: a Spanish cohort simulation

**Journal:** PLOS One

Manuscript No.: PONE-D-25-64496

**Corresponding Author:** Juan Cosin Sales, jcosinsales@gmail.com

Dear PLOS One Academic Editor, Prof. Paolo Magni,

We are grateful for the opportunity to revise and resubmit our manuscript to PLOS ONE. We would like to sincerely thank you and both reviewers for the thorough and constructive review of our work. Their comments have enabled us to strengthen the manuscript and its scientific argumentation. We have addressed all journal requirements and reviewer comments below, point by point.

Please find attached:

1. A response to reviewers letter (this document).
2. A revised manuscript with track changes (highlighting all changes made).
3. A clean manuscript without tracked changes.

## Comments regarding journal requirements

#### Requirement 1 — PLOS ONE Style Formatting

**Response**: We have reviewed the PLOS ONE style requirements for manuscript formatting and have ensured that the revised manuscript is consistent with the PLOS ONE templates for main body, title, and author affiliations. We have updated the manuscript accordingly.

#### Requirement 2 — Code Sharing

**Response**: The present study is based on a Monte Carlo simulation implemented in SAS version 9.2. The analysis code consists of SAS scripts used to apply published drug efficacy parameters (from Toth et al. 2022 NMA) to individual patient-level LDL-C values from the IQVIA EMR database. In compliance with PLOS One's code-sharing policy, the author-generated SAS code will be made fully available upon publication via a public repository (GitHub/Zenodo), without restrictions. We have updated the Data Availability Statement accordingly.

#### Requirements 3 & 4 — Competing Interests and Funding Statement

**Response**: We thank the Editor for highlighting the need for an expanded and explicit statement. We provide the following updated statements:

**Updated Funding Statement:**

"This study was supported by Daiichi Sankyo Spain. The funder provided financial support for study design, data acquisition, and medical writing support provided by IQVIA. One author (E.A.I.) is an employee of Daiichi Sankyo Spain and contributed to the preparation and revision of the manuscript. The remaining authors independently conducted data analysis and prepared the manuscript. The specific roles of all authors are articulated in the 'author contributions' section.”

**Updated Competing Interests Statement:**

"Juan Cosin-Sales has received personal fees for consulting and/or speaking engagements from Daiichi Sankyo, Amgen, Sanofi, MSD, Amarin, Ferrer, and Novartis, outside the submitted work. José María Mostaza Prieto has received consulting and/or speaker honoraria from Daiichi Sankyo, Novartis, Amgen, Sanofi, Ultragenyx, Ferrer, Servier, and Alter, outside the submitted work. José Antonio Martín-Conde has received honoraria from Alnylam for educational activities, outside the submitted work, and research support from Daiichi Sankyo for the present study. Maria Reyes Abad-Sazatornil has received consulting fees from CINFA and honoraria for lectures or educational events from Novartis, AstraZeneca, Johnson & Johnson, GSK, UCB, Ascendis Pharma, and SOBI, as well as support for attending meetings from Roche, Pfizer, and Takeda, all outside the submitted work. Eder Alonso Iglesias is an employee of Daiichi Sankyo Spain; no personal fees were received for the writing of this article. Mafalda Carmo and Aaron Aires are employees of IQVIA, which received funding from Daiichi Sankyo Spain to provide medical writing support for this study; no personal fees were received by these authors for the writing of this article. No other author received personal fees for writing this article. All authors have completed the ICMJE disclosure form. This does not alter our adherence to PLOS ONE policies on sharing data and materials."

#### Requirements 5 & 6 — Data Availability

**Response**: We acknowledge PLOS One's data sharing policy. The patient-level data used in this study are derived from the IQVIA Electronic Medical Record (EMR) database, which is a proprietary, commercially licensed database containing longitudinal anonymized real-world data. These data are owned by IQVIA and cannot be made publicly available due to data governance and commercial licensing restrictions. Data access requests may be directed to IQVIA (Spain) at the following contact: mafalda.carmo@iqvia.com. Summary statistics, simulation parameters, and the data underlying all figures and tables are included in the manuscript and its Supporting Information files. The SAS analysis scripts will be made publicly available via GitHub/Zenodo upon acceptance. We have updated the Data Availability Statement accordingly.

#### Requirement 7 — Figure S1 Resolution

**Response**: We thank the Editor for this remark. We have uploaded a new, higher-resolution version of Figure S1 to better illustrate the dispersion of LDL-C values per patient at each step, in compliance with PLOS One's figure guidelines. We have also added 3 additional complementing figures to better illustrate the patient flow (S2, S3, S4).

#### Requirement 8 — Supporting Information File Type

**Response**: We have amended the file type of Figure S1 from "Figure" to "Supporting Information" as requested. We have also confirmed that a legend for the figures is included in the manuscript text, after the References section.

## Comments from reviewer 1

#### **General comment:** *"This is a technically sound, policy-relevant Monte Carlo simulation study using a large real-world Spanish cohort (n=34,967 uncontrolled high/VHR patients). It directly addresses a current gap in the Spanish lipid-management algorithm (2024 SEC/SEA consensus recommending BA before PCSK9i/INC) and demonstrates clear budget-impact advantages (~28–30% cost reduction) with near-identical target attainment (98%/88%). The approach mirrors and extends the published German simulation (Katzmann et al., PLoS One 2022), adding inclisiran and Spain-specific pricing/reimbursement context."*

**Response:** We sincerely thank the Reviewer for the thorough and detailed assessment of our manuscript, and for the kind recognition that the study is technically sound, policy-relevant, and a meaningful extension of the existing evidence base. We are also grateful for the specific and constructive suggestions, which we address below point by point.

#### **Comment 1:** *"Justify applicability or replace with a more representative rate for Spanish/European high/VHR populations (literature suggests ~1.5–2.7% annual in mixed high-risk cohorts; e.g., UKPDS/CALIBER-derived rates ~2.5% in T2DM)."*

**Response:** We thank the Reviewer for this comment. The baseline 4P-MACE rate of 3.4% used in the primary analysis was sourced from Lee et al. (2017, Rev Esp Cardiol). We agree that, as this source reported event rates from clinical trials with a study population comprising predominantly of post-stent coronary artery disease patients, a higher-acuity group than the heterogeneous real-world HR/VHR patients in our IQVIA EMR cohort, it is not the most adequate source and removed this reference.

To the best of our knowledge, no single published study reports a directly applicable annual 4P-MACE rate for our population. Our clinical experience and available evidence suggest 3.0%/year is a plausible assumption: annual 3P-MACE rates of 2.5–2.6%/year have been reported in real-world T2DM cohorts (e.g. Martín-Enguix, 2025), rising to 6.5%/year in Spanish patients with DM and established coronary syndrome (Mateos de la Haba, 2022, CICCOR study). Our assumption sits within this range, reflecting our mixed HR/VHR population (71% VHR, 39.2% ASCVD, 37.1% DM, 39.2% with atherosclerotic cardiovascular disease). We have updated the primary analysis, accordingly, as agreed with the clinical expert co-authors. More fundamentally, the baseline rate does not affect the relative comparison between strategies, as the CTTC-derived RRR applies proportionally in both arms. The sensitivity analysis across 2.0%–4.0% (S7 Table) confirms that all directional conclusions are fully robust regardless of the rate assumed.

#### **Comment 2**: "Perform and report sensitivity analyses using alternative baselines (e.g., 2%, 2.5%, 4%)."

**Response**: We thank the Reviewer for this suggestion, which we have fully implemented. We have conducted a linear sensitivity analysis of prevented 4P-MACE events across five baseline annual event rates: 2.0%, 2.5%, 3.0% (primary analysis), 3.5%, and 4.0%. Results are reported in the new Supplementary Table S7. The results confirm that the directional conclusions are robust across the entire range cited by the Reviewer: regardless of the baseline rate assumed, the BA-inclusive strategy consistently prevents fewer MACE than direct PCSK9i escalation (by 0.20–0.46 events per 100 patients/year), and this difference scales proportionally with the assumed rate. The absolute difference between strategies remains modest across all scenarios, further supporting the Discussion framing that the clinical trade-off is small relative to the substantial cost savings.

#### Comment 3: "Clarify whether the RRR (21% per 1 mmol/L LDL-C reduction from CTTC) is applied to the absolute LDL-C reduction from the post-EZE level (or from untreated baseline) and how 'prevented events' are computed differentially between BA-inclusive vs. BA-free arms."

**Response:** We thank the Reviewer for requesting this important clarification, which we have now addressed explicitly in the Methods section.

The 21% RRR per 1 mmol/L (38.67 mg/dL) LDL-C reduction (CTTC, Lancet 2010) is applied sequentially at each step of the simulation, consistently from the LDL-C level at the start of each treatment step (i.e., not from the untreated baseline), in a manner methodologically consistent with Katzmann et al. (2022). Specifically, prevented events are computed across three sequential steps, both arms sharing Step 1:

- **Step 1, EZE step**: The RRR is applied to the mean LDL-C reduction achieved by EZE simulation across the full cohort (N=34,967). For patients not yet on EZE (n=31,501), this reflects the reduction from their baseline LDL-C to the post-EZE simulated level; for patients already on EZE (n=3,466), no additional LDL-C reduction is attributed to EZE. The effective mean LDL-C reduction across the full cohort is used to compute events prevented at this step. This ensures that EZE-mediated MACE prevention is captured for all newly EZE-simulated patients.
- **Step 2, BA step:** For patients entering the BA simulation and achieving LDL-C control (n=11,167; 38.3% of BA entrants), the RRR is applied to the LDL-C reduction from the post-EZE level to the post-BA level.
- **Step 3, E/A or INC step:** For patients not controlled at BA (n=17,977), the BA LDL-C effect is reversed to the post-EZE level before E/A or INC simulation, and the RRR reflects only the E/A or INC reduction from that post-EZE level. In the Comparator arm, the RRR is applied to the E/A or INC reduction from the post-EZE level for all BA-eligible patients (n=29,144).

The differential in prevented events between arms arises because direct E/A or INC escalation achieves a greater absolute LDL-C reduction than BA followed by E/A or INC (since the latter group starts PCSK9i from a lower LDL-C baseline due to partial BA response in controlled patients, but the non-controlled patients start from the same post-EZE level in both arms), resulting in more prevented MACE in the Comparator arm, a trade-off that comes at higher cost.

We have updated this section of the manuscript to clarify readers.

#### **Comment 4:** "Explicitly state in Limitations that the small difference (0.3–0.4 events per 100 patients/year) is sensitive to these assumptions and that long-term modeling (beyond 1 year) with discounting would be valuable for future work."

**Response**: We thank the Reviewer and have added the following to the Limitations section:

*"The estimated difference in prevented 4P-MACE events between BA-inclusive and BA-free strategies (0.2–0.5 events per 100 patients per year across the range of plausible baseline incidence rates tested in sensitivity analyses) is modest and sensitive to the assumed baseline annual event rate and the CTTC-derived RRR per mmol/L LDL-C reduction. Furthermore, the present model is limited to a 1-year time horizon. Long-term modelling incorporating time-discounting, treatment discontinuation, and event-free survival beyond 12 months would provide a more complete health-economic picture and is recommended for future work."*

#### **Comment 5:** "Explicitly note that actual NHS acquisition costs (often discounted via tenders) differ, especially since PCSK9i/inclisiran are fully reimbursed while bempedoic acid reimbursement is more restricted."

**Response**: We thank the Reviewer for this observation. We have added the following to the Limitations section:

*"Cost estimates are based on official list prices published by the Spanish Ministry of Health, as recommended by the CAPF/GENESIS pharmacoeconomic evaluation guidelines. In practice, the Spanish NHS may acquire drugs at negotiated prices below list, and BA, E/A, and INC are all reimbursed under restricted indications and special funding conditions, such as expenditure ceilings, which affect the final cost to the NHS. Furthermore, the simulation did not account for current Spanish reimbursement criteria for PCSK9i, which restrict eligibility to patients with LDL-C >100 mg/dL, well above ESC guideline targets, meaning the benefit of BA would likely be greater if these thresholds were applied, as BA would represent the only escalation option for many patients after statin and EZE."*

#### **Comment 6:** "The savings estimate (~€1,174–1,679 per patient/year) therefore reflects a payer perspective using list prices; real-world NHS savings may be higher (or lower) depending on contracts."

**Response**: We thank the Reviewer for this observation, which is closely related to Comment 5. We confirm and have now explicitly stated in the Methods (Budget Impact section) that the cost analysis reflects a retail list price perspective, consistent with Spanish pharmacoeconomic evaluation guidelines. We have added:

"*All costs reflect official retail prices and represent a payer perspective based on list prices. Real-world NHS acquisition costs may differ due to negotiated discounts or risk-sharing agreements, and the present estimates should be interpreted accordingly*."

#### **Comment 7:** "For inclisiran, confirm use of only the maintenance-year cost (€4,696) and add a sensitivity analysis using first-year cost (€7,045)."

**Response**: We confirm that only the maintenance-year cost (€4,696) was used in the base-case analysis. This is a deliberate methodological choice to avoid overestimating savings in the first year and to align with prior simulation studies. We have added a new sensitivity analysis (Supplementary Table S8) using the first-year inclisiran cost (€7,045). Results confirm that using first-year inclisiran costs would further increase the estimated savings from the BA-inclusive strategy (-30.7% vs -27.2%). These percentages reflect the relative reduction in annual treatment cost per patient across the full simulated cohort (N=34,967), weighted by the proportion of patients reaching each treatment step — that is, they are not limited to patients who ultimately received INC, but account for the entire treatment sequence including those controlled at EZE or BA and therefore not escalated to INC. As a result, the weighted average cost per patient is lower than the per-patient INC cost, and the relative savings from incorporating BA before INC reflect the full budget impact across all simulated patients.

#### **Comment 8:** "Limitations Section – Expand Slightly. Add: (a) geographic coverage limited to three regions in the IQVIA EMR (~3% of Spanish population, though demographically representative); (b) no modeling of adherence, discontinuation, or titration delays (real-world persistence with oral BA may exceed injectables); (c) uniform application of NMA efficacy across subgroups (no interaction testing by baseline LDL-C, statin intensity, or risk category)."

**Response**: We have expanded the Limitations section to address each point explicitly:

**(a)** "*The IQVIA EMR database covers three health regions in Spain, representing approximately 3% of the national population (~1.2 million patients). While prior studies have confirmed its demographic representativeness of the broader Spanish population, generalizability to all Spanish regions should be interpreted with appropriate caution.*"

(b) " *The simulation model assumes optimal adherence and does not account for real-world discontinuation rates, titration delays, or differences in persistence across therapies. In clinical practice, oral therapies such as BA may demonstrate higher long-term adherence compared with injectable regimens due to the convenience of once-daily oral administration, while bi-annual injectable regimens (INC) may benefit from higher persistence once initiated. These adherence differences could meaningfully affect long-term cost and clinical outcomes.*"

(c) "*Efficacy parameters from the Toth et al. (2022) NMA were applied uniformly across the simulation cohort, without stratification by baseline LDL-C level, statin intensity, or cardiovascular risk category, which may not fully capture the heterogeneity of LDL-C response observed in clinical practice.*"

## REVIEWER #2

#### **General assessment:** "In Spain, the introduction of bempedoic acid before injection therapy has proven to be a clinically effective treatment option for LDL-C management in patients at high risk of cardiovascular events, and it has been reported to contribute to reducing medical costs while maintaining good LDL-C control rates. The clinical implications of this study are likely to be beneficial, but I would like to see further investigation into whether there are differences in LDL-C management and cost reduction effects between those with and without diabetes."

**Response**: We sincerely thank the Reviewer for the positive assessment and for the important question regarding potential differences in LDL-C management and cost reduction between patients with and without diabetes mellitus (DM).

DM patients comprise 37.1% of our simulation cohort and are overrepresented in the VHR stratum (42.1% vs. 24.7% in HR), reflecting that DM with additional risk factors drives VHR classification per 2021 ESC guidelines. Regarding BA specifically:

- A CLEAR Outcomes sub analysis (Ray et al., Lancet Diabetes Endocrinol, 2024) demonstrated significant and consistent MACE-4 reduction with BA in patients with DM, with no evidence of effect modification across glycemic strata.
- Unlike statins, BA does not increase HbA1c levels or new-onset diabetes risk (Leiter et al., Diabetes Obes Metab, 2022), a clinical advantage in this subgroup.

Since no DM-stratified analyses were pre-specified or conducted for this study, performing a post-hoc subgroup analysis is beyond the scope of this revision. However, we have added the following to the Discussion:

*"A clinically relevant subgroup for future investigation is patients with diabetes mellitus (DM), who comprised 37.1% of our simulation cohort and are overrepresented in the very-high-risk stratum (42.1%). Evidence suggests that bempedoic acid provides significant and consistent LDL-C reduction and MACE-4 risk reduction across glycemic strata, with potentially greater absolute benefit in patients with DM given their higher baseline cardiovascular risk, and without increasing HbA1c levels or new-onset diabetes risk. Future simulation studies should consider stratified analyses by diabetes status to quantify the differential impact on LDL-C management and cost reduction in this high-risk subgroup.*"

We have also added to the Limitations:

" *Subgroup analyses by diabetes status, sex, or statin intensity were not performed. Given the high prevalence of DM in this cohort (37.1%) and emerging evidence of consistent or greater BA benefit in this subgroup, stratified analyses are recommended for future work."*

## Table of changes to the manuscript

All changes are marked using track changes in the word document. The table below lists the changes:

| **Section** | **Change made** |
| --- | --- |
| Methods: Prevented MACE | Updated baseline rate from 3.4% to 3.0%; removed Lee 2017 reference; added new Spanish references; clarified the RRR sequential application |
| Methods, Budget impact | Added list price statement; confirmed INC maintenance cost; added S8 Table with sensitivity analysis |
| Results, MACE | Updated figures to 0.35 and 0.30 per 100 patients (using the 3% assumption) |
| Discussion | Added DM subgroup paragraph |
| Limitations | Expanded considering the different aspects mentioned by reviewers such as geographic representativeness, adherence, uniform NMA efficacy, DM subgroup, INC first-year cost, sensitivity to MACE rates |
| Supporting Information | Added S2–S4 (patient flow), S5 (cohort flow), S6 (mean LDL), S7 (MACE sensitivity), S8 (INC cost sensitivity) |
| Figure S1 | Re-uploaded at higher resolution (new figure style, same data) |
| Data Availability | Updated for IQVIA restrictions and code sharing |
| Funding & COI | Updated per PLOS ONE requirements |

We hope that the revised manuscript and these responses satisfactorily address all concerns raised by the Editor and both Reviewers. We remain available to provide any further clarification or information that may be required.

We would like to reiterate our sincere gratitude to the Editorial team of PLOS ONE and to both reviewers for the time and care dedicated to the review of our manuscript. Their constructive input has materially improved the quality and transparency of the work.

Yours sincerely,

**Juan Cosin Sales**

*Head of Cardiology, Hospital Arnau de Vilanova*

*Director, Research Agency of the Spanish Society of Cardiology*

*Associate Professor, CEU Cardenal Herrera University*

*On behalf of all authors*
