# Supplementary material for: LDL-C target attainment and treatment costs in high-risk and very-high-risk patients with or without bempedoic acid: A Spanish cohort simulation
Source: PLoS One. 2026 Aug 14;21(8):e0353193. doi: 10.1371/journal.pone.0353193 (PMC13475820; doi:10.1371/journal.pone.0353193)
Supplement: Peer review history

# Peer review: Decision Letter 0

**DOI:** 10.1371/journal.pone.0353193.r001

**Authors:** Paolo Magni

19 Mar 2026

Dear Dr. Cosin Sales,

Please submit your revised manuscript by May 03 2026 11:59PM. If you will need more time than this to complete your revisions, please reply to this message or contact the journal office at plosone@plos.org. When you're ready to submit your revision, log on to https://www.editorialmanager.com/pone/ and select the 'Submissions Needing Revision' folder to locate your manuscript file.

- A letter that responds to each point raised by the academic editor and reviewer(s). You should upload this letter as a separate file labeled 'Response to Reviewers'.
- A marked-up copy of your manuscript that highlights changes made to the original version. You should upload this as a separate file labeled 'Revised Manuscript with Track Changes'.
- An unmarked version of your revised paper without tracked changes. You should upload this as a separate file labeled 'Manuscript'.

If you would like to make changes to your financial disclosure, please include your updated statement in your cover letter. Guidelines for resubmitting your figure files are available below the reviewer comments at the end of this letter.

If applicable, we recommend that you deposit your laboratory protocols in protocols.io to enhance the reproducibility of your results. Protocols.io assigns your protocol its own identifier (DOI) so that it can be cited independently in the future. For instructions see: https://journals.plos.org/plosone/s/submission-guidelines#loc-laboratory-protocols. Additionally, PLOS ONE offers an option for publishing peer-reviewed Lab Protocol articles, which describe protocols hosted on protocols.io. Read more information on sharing protocols at https://plos.org/protocols?utm_medium=editorial-email&utm_source=authorletters&utm_campaign=protocols.

We look forward to receiving your revised manuscript.

Kind regards,

Paolo Magni

Academic Editor

PLOS One

Journal Requirements:

When submitting your revision, we need you to address these additional requirements.

1. Please ensure that your manuscript meets PLOS ONE's style requirements, including those for file naming. The PLOS ONE style templates can be found at

https://journals.plos.org/plosone/s/file?id=wjVg/PLOSOne_formatting_sample_main_body.pdf and https://journals.plos.org/plosone/s/file?id=ba62/PLOSOne_formatting_sample_title_authors_affiliations.pdf

2. Please note that PLOS One has specific guidelines on code sharing for submissions in which author-generated code underpins the findings in the manuscript. In these cases, all author-generated code must be made available without restrictions upon publication of the work. Please review our guidelines at https://journals.plos.org/plosone/s/materials-and-software-sharing#loc-sharing-code and ensure that your code is shared in a way that follows best practice and facilitates reproducibility and reuse.

3. Thank you for stating the following in the Competing Interests and Financial Disclosure sections:

[The authors thank Daiichi Sankyo for supporting the present study. Juan Cosin Sales has received personal fees for consulting and/or speaking engagements from Daiichi Sankyo, Amgen, Sanofi, MSD, Amaryn, Ferrer, and Novartis. José María Mostaza Prieto has received consulting and/or speaker honoraria from Daiichi Sankyo, Novartis, Amgen, Sanofi, Ultragenyx, Ferrer, Servier, and Alter. José Antonio Martín Conde has received support from Daiichi Sankyo for the present manuscript and honoraria from Alnylam for educational activities. Maria Reyes Abad-Sazatornil has received consulting fees from CINFA and honoraria for lectures or educational events from Novartis, AstraZeneca, Johnson & Johnson, GSK, UCB, Ascendis Pharma, and SOBI, as well as support for attending meetings from Roche, Pfizer, and Takeda. Eder Alonso Iglesias is an employee of Daiichi Sankyo. All authors have completed the ICMJE disclosure form and declare that these relationships have been transparently reported.].

We note that you received funding from these commercial sources, and that one or more of the authors have an affiliation and/or employed to the commercial funders of this research study: Daiichi Sankyo, Amgen, Sanofi, MSD, Amarin, Ferrer, Novartis, Ultragenyx, Servier, Alter, Alnylam, CINFA, AstraZeneca, Johnson & Johnson, GSK, UCB, Ascendis Pharma, SOBI, Roche, Pfizer, Takeda, IQVIA, and Daiichi Sankyo Europe GmbH.

1. Please provide an amended Funding Statement declaring this commercial affiliation, as well as a statement regarding the Role of Funders in your study. If the funding organization did not play a role in the study design, data collection and analysis, decision to publish, or preparation of the manuscript and only provided financial support in the form of authors' salaries and/or research materials, please review your statements relating to the author contributions, and ensure you have specifically and accurately indicated the role(s) that these authors had in your study. You can update author roles in the Author Contributions section of the online submission form.

Please also include the following statement within your amended Funding Statement.

“The funder provided support in the form of salaries for authors [insert relevant initials], but did not have any additional role in the study design, data collection and analysis, decision to publish, or preparation of the manuscript. The specific roles of these authors are articulated in the ‘author contributions’ section.”

If your commercial affiliation did play a role in your study, please state and explain this role within your updated Funding Statement.

2. Please also provide an updated Competing Interests Statement declaring this commercial affiliation along with any other relevant declarations relating to employment, consultancy, patents, products in development, or marketed products, etc.

Within your Competing Interests Statement, please confirm that this commercial affiliation does not alter your adherence to all PLOS ONE policies on sharing data and materials by including the following statement: "This does not alter our adherence to PLOS ONE policies on sharing data and materials.” (as detailed online in our guide for authors http://journals.plos.org/plosone/s/competing-interests). If this adherence statement is not accurate and there are restrictions on sharing of data and/or materials, please state these. Please note that we cannot proceed with consideration of your article until this information has been declared.

Please include both an updated Funding Statement and Competing Interests Statement in your cover letter. We will change the online submission form on your behalf.

4. Thank you for stating the following financial disclosure:

[The authors thank Daiichi Sankyo for supporting the present study. Juan Cosin Sales has received personal fees for consulting and/or speaking engagements from Daiichi Sankyo, Amgen, Sanofi, MSD, Amaryn, Ferrer, and Novartis. José María Mostaza Prieto has received consulting and/or speaker honoraria from Daiichi Sankyo, Novartis, Amgen, Sanofi, Ultragenyx, Ferrer, Servier, and Alter. José Antonio Martín Conde has received support from Daiichi Sankyo for the present manuscript and honoraria from Alnylam for educational activities. Maria Reyes Abad-Sazatornil has received consulting fees from CINFA and honoraria for lectures or educational events from Novartis, AstraZeneca, Johnson & Johnson, GSK, UCB, Ascendis Pharma, and SOBI, as well as support for attending meetings from Roche, Pfizer, and Takeda. Eder Alonso Iglesias is an employee of Daiichi Sankyo. All authors have completed the ICMJE disclosure form and declare that these relationships have been transparently reported.].

Please state what role the funders took in the study.  If the funders had no role, please state: "The funders had no role in study design, data collection and analysis, decision to publish, or preparation of the manuscript."

If this statement is not correct you must amend it as needed.

Please include this amended Role of Funder statement in your cover letter; we will change the online submission form on your behalf.

5. We note that you have indicated that there are restrictions to data sharing for this study. For studies involving human research participant data or other sensitive data, we encourage authors to share de-identified or anonymized data. However, when data cannot be publicly shared for ethical reasons, we allow authors to make their data sets available upon request. For information on unacceptable data access restrictions, please see http://journals.plos.org/plosone/s/data-availability#loc-unacceptable-data-access-restrictions.

Before we proceed with your manuscript, please address the following prompts:

a) If there are ethical or legal restrictions on sharing a de-identified data set, please explain them in detail (e.g., data contain potentially identifying or sensitive patient information, data are owned by a third-party organization, etc.) and who has imposed them (e.g., a Research Ethics Committee or Institutional Review Board, etc.). Please also provide contact information for a data access committee, ethics committee, or other institutional body to which data requests may be sent.

b) If there are no restrictions, please upload the minimal anonymized data set necessary to replicate your study findings to a stable, public repository and provide us with the relevant URLs, DOIs, or accession numbers. Please see http://www.bmj.com/content/340/bmj.c181.long for guidelines on how to de-identify and prepare clinical data for publication. For a list of recommended repositories, please see https://journals.plos.org/plosone/s/recommended-repositories. You also have the option of uploading the data as Supporting Information files, but we would recommend depositing data directly to a data repository if possible.

Please update your Data Availability statement in the submission form accordingly.

6. We note that your Data Availability Statement is currently as follows: [Relevant data are within the manuscript and its Supporting Information files.]

Please confirm at this time whether or not your submission contains all raw data required to replicate the results of your study. Authors must share the “minimal data set” for their submission. PLOS defines the minimal data set to consist of the data required to replicate all study findings reported in the article, as well as related metadata and methods (https://journals.plos.org/plosone/s/data-availability#loc-minimal-data-set-definition).

For example, authors should submit the following data:

- The values behind the means, standard deviations and other measures reported;

- The values used to build graphs;

- The points extracted from images for analysis.

Authors do not need to submit their entire data set if only a portion of the data was used in the reported study.

If your submission does not contain these data, please either upload them as Supporting Information files or deposit them to a stable, public repository and provide us with the relevant URLs, DOIs, or accession numbers. For a list of recommended repositories, please see https://journals.plos.org/plosone/s/recommended-repositories.

If there are ethical or legal restrictions on sharing a de-identified data set, please explain them in detail (e.g., data contain potentially sensitive information, data are owned by a third-party organization, etc.) and who has imposed them (e.g., an ethics committee). Please also provide contact information for a data access committee, ethics committee, or other institutional body to which data requests may be sent. If data are owned by a third party, please indicate how others may request data access.

7. Please upload a new copy of Figure S1 as the detail is not clear. Please follow the link for more information:  https://journals.plos.org/plosone/s/figures

8. We notice that your supplementary figures are uploaded with the file type “Figure”. Please amend the file type to 'Supporting Information'. Please ensure that each Supporting Information file has a legend listed in the manuscript after the references list.

If the reviewer comments include a recommendation to cite specific previously published works, please review and evaluate these publications to determine whether they are relevant and should be cited. There is no requirement to cite these works unless the editor has indicated otherwise.

Additional Editor Comments:

The paper reports an interesting study. But all the issues raised by the Reviwers need to be addressed in full.

[Note: HTML markup is below. Please do not edit.]

Reviewers' comments:

Reviewer's Responses to Questions

**Comments to the Author**

1. Is the manuscript technically sound, and do the data support the conclusions?

Reviewer #1: Yes

Reviewer #2: Yes

**********

2. Has the statistical analysis been performed appropriately and rigorously?

Reviewer #1: Yes

Reviewer #2: I Don't Know

**********

3. Have the authors made all data underlying the findings in their manuscript fully available?

The PLOS Data policy

Reviewer #1: Yes

Reviewer #2: Yes

**********

4. Is the manuscript presented in an intelligible fashion and written in standard English?

Reviewer #1: Yes

Reviewer #2: Yes

**********

Reviewer #1: This is a technically sound, policy-relevant Monte Carlo simulation study using a large real-world Spanish cohort (n=34,967 uncontrolled high/VHR patients). It directly addresses a current gap in the Spanish lipid-management algorithm (2024 SEC/SEA consensus recommending BA before PCSK9i/INC) and demonstrates clear budget-impact advantages (~28–30% cost reduction) with near-identical target attainment (98%/88%). The approach mirrors and extends the published German simulation (Katzmann et al., PLoS One 2022), adding inclisiran and Spain-specific pricing/reimbursement context.

1. Justify applicability or replace with a more representative rate for Spanish/European high/VHR populations (literature suggests ~1.5–2.7% annual in mixed high-risk cohorts; e.g., UKPDS/CALIBER-derived rates ~2.5% in T2DM).

2. Perform and report sensitivity analyses using alternative baselines (e.g., 2%, 2.5%, 4%).

3. Clarify whether the RRR (21% per 1 mmol/L LDL-C reduction from CTTC) is applied to the absolute LDL-C reduction from the post-EZE level (or from untreated baseline) and how "prevented events" are computed differentially between BA-inclusive vs. BA-free arms.

4. Explicitly state in Limitations that the small difference (0.3–0.4 events per 100 patients/year) is sensitive to these assumptions and that long-term modeling (beyond 1 year) with discounting would be valuable for future work.

5. Explicitly note that actual NHS acquisition costs (often discounted via tenders) differ, especially since PCSK9i/inclisiran are fully reimbursed while bempedoic acid reimbursement is more restricted.

6. The savings estimate (~€1,174–1,679 per patient/year) therefore reflects a payer perspective using list prices; real-world NHS savings may be higher (or lower) depending on contracts.

7. For inclisiran, confirm use of only the maintenance-year cost (€4,696) and add a sensitivity analysis using first-year cost (€7,045).

8. Limitations Section – Expand Slightly

Add: (a) geographic coverage limited to three regions in the IQVIA EMR (~3% of Spanish population, though demographically representative); (b) no modeling of adherence, discontinuation, or titration delays (real-world persistence with oral BA may exceed injectables); (c) uniform application of NMA efficacy across subgroups (no interaction testing by baseline LDL-C, statin intensity, or risk category).

Reviewer #2: In Spain, the introduction of bempedoic acid before injection therapy has proven to be a clinically effective treatment option for LDL-C management in patients at high risk of cardiovascular events, and it has been reported to contribute to reducing medical costs while maintaining good LDL-C control rates.

The clinical implications of this study are likely to be beneficial, but I would like to see further investigation into whether there are differences in LDL-C management and cost reduction effects between those with and without diabetes.

**********

what does this mean?). If published, this will include your full peer review and any attached files.

If you choose “no”, your identity will remain anonymous but your review may still be made public.

**Do you want your identity to be public for this peer review?**  For information about this choice, including consent withdrawal, please see our Privacy Policy

Reviewer #1: No

Reviewer #2: **Yes:**  Ken-ichi Aihara

**********

[NOTE: If reviewer comments were submitted as an attachment file, they will be attached to this email and accessible via the submission site. Please log into your account, locate the manuscript record, and check for the action link "View Attachments". If this link does not appear, there are no attachment files.]

To ensure your figures meet our technical requirements, please review our figure guidelines: https://journals.plos.org/plosone/s/figures

You may also use PLOS’s free figure tool, NAAS, to help you prepare publication quality figures: https://journals.plos.org/plosone/s/figures#loc-tools-for-figure-preparation.

NAAS will assess whether your figures meet our technical requirements by comparing each figure against our figure specifications.

# Author comment: Author response to Decision Letter 1

**DOI:** 10.1371/journal.pone.0353193.r002

6 May 2026

Dear PLOS One Academic Editor, Prof. Paolo Magni,

We are grateful for the opportunity to revise and resubmit our manuscript to PLOS ONE. We would like to sincerely thank you and both reviewers for the thorough and constructive review of our work. Their comments have enabled us to strengthen the manuscript and its scientific argumentation. We have addressed all journal requirements and reviewer comments below, point by point.

Please find attached:

1. A response to reviewers letter (this document).

2. A revised manuscript with track changes (highlighting all changes made).

3. A clean manuscript without tracked changes.

Comments regarding journal requirements

Requirement 1 — PLOS ONE Style Formatting

Response: We have reviewed the PLOS ONE style requirements for manuscript formatting and have ensured that the revised manuscript is consistent with the PLOS ONE templates for main body, title, and author affiliations. We have updated the manuscript accordingly.

Requirement 2 — Code Sharing

Response: The present study is based on a Monte Carlo simulation implemented in SAS version 9.2. The analysis code consists of SAS scripts used to apply published drug efficacy parameters (from Toth et al. 2022 NMA) to individual patient-level LDL-C values from the IQVIA EMR database. In compliance with PLOS One's code-sharing policy, the author-generated SAS code will be made fully available upon publication via a public repository (GitHub/Zenodo), without restrictions. We have updated the Data Availability Statement accordingly.

Requirements 3 & 4 — Competing Interests and Funding Statement

Response: We thank the Editor for highlighting the need for an expanded and explicit statement. We provide the following updated statements:

Updated Funding Statement:

"This study was supported by Daiichi Sankyo Spain. The funder provided financial support for study design, data acquisition, and medical writing support provided by IQVIA. One author (E.A.I.) is an employee of Daiichi Sankyo Spain and contributed to the preparation and revision of the manuscript. The remaining authors independently conducted data analysis and prepared the manuscript. The specific roles of all authors are articulated in the 'author contributions' section.”

Updated Competing Interests Statement:

"Juan Cosin-Sales has received personal fees for consulting and/or speaking engagements from Daiichi Sankyo, Amgen, Sanofi, MSD, Amarin, Ferrer, and Novartis, outside the submitted work. José María Mostaza Prieto has received consulting and/or speaker honoraria from Daiichi Sankyo, Novartis, Amgen, Sanofi, Ultragenyx, Ferrer, Servier, and Alter, outside the submitted work. José Antonio Martín-Conde has received honoraria from Alnylam for educational activities, outside the submitted work, and research support from Daiichi Sankyo for the present study. Maria Reyes Abad-Sazatornil has received consulting fees from CINFA and honoraria for lectures or educational events from Novartis, AstraZeneca, Johnson & Johnson, GSK, UCB, Ascendis Pharma, and SOBI, as well as support for attending meetings from Roche, Pfizer, and Takeda, all outside the submitted work. Eder Alonso Iglesias is an employee of Daiichi Sankyo Spain; no personal fees were received for the writing of this article. Mafalda Carmo and Aaron Aires are employees of IQVIA, which received funding from Daiichi Sankyo Spain to provide medical writing support for this study; no personal fees were received by these authors for the writing of this article. No other author received personal fees for writing this article. All authors have completed the ICMJE disclosure form. This does not alter our adherence to PLOS ONE policies on sharing data and materials."

Requirements 5 & 6 — Data Availability

Response: We acknowledge PLOS One's data sharing policy. The patient-level data used in this study are derived from the IQVIA Electronic Medical Record (EMR) database, which is a proprietary, commercially licensed database containing longitudinal anonymized real-world data. These data are owned by IQVIA and cannot be made publicly available due to data governance and commercial licensing restrictions. Data access requests may be directed to IQVIA (Spain) at the following contact: mafalda.carmo@iqvia.com. Summary statistics, simulation parameters, and the data underlying all figures and tables are included in the manuscript and its Supporting Information files. The SAS analysis scripts will be made publicly available via GitHub/Zenodo upon acceptance. We have updated the Data Availability Statement accordingly.

Requirement 7 — Figure S1 Resolution

Response: We thank the Editor for this remark. We have uploaded a new, higher-resolution version of Figure S1 to better illustrate the dispersion of LDL-C values per patient at each step, in compliance with PLOS One's figure guidelines. We have also added 3 additional complementing figures to better illustrate the patient flow (S2, S3, S4).

Requirement 8 — Supporting Information File Type

Response: We have amended the file type of Figure S1 from "Figure" to "Supporting Information" as requested. We have also confirmed that a legend for the figures is included in the manuscript text, after the References section.

Comments from reviewer 1

General comment: "This is a technically sound, policy-relevant Monte Carlo simulation study using a large real-world Spanish cohort (n=34,967 uncontrolled high/VHR patients). It directly addresses a current gap in the Spanish lipid-management algorithm (2024 SEC/SEA consensus recommending BA before PCSK9i/INC) and demonstrates clear budget-impact advantages (~28–30% cost reduction) with near-identical target attainment (98%/88%). The approach mirrors and extends the published German simulation (Katzmann et al., PLoS One 2022), adding inclisiran and Spain-specific pricing/reimbursement context."

Response: We sincerely thank the Reviewer for the thorough and detailed assessment of our manuscript, and for the kind recognition that the study is technically sound, policy-relevant, and a meaningful extension of the existing evidence base. We are also grateful for the specific and constructive suggestions, which we address below point by point.

Comment 1: "Justify applicability or replace with a more representative rate for Spanish/European high/VHR populations (literature suggests ~1.5–2.7% annual in mixed high-risk cohorts; e.g., UKPDS/CALIBER-derived rates ~2.5% in T2DM)."

Response: We thank the Reviewer for this comment. The baseline 4P-MACE rate of 3.4% used in the primary analysis was sourced from Lee et al. (2017, Rev Esp Cardiol). We agree that, as this source reported event rates from clinical trials with a study population comprising predominantly of post-stent coronary artery disease patients, a higher-acuity group than the heterogeneous real-world HR/VHR patients in our IQVIA EMR cohort, it is not the most adequate source and removed this reference.

To the best of our knowledge, no single published study reports a directly applicable annual 4P-MACE rate for our population. Our clinical experience and available evidence suggest 3.0%/year is a plausible assumption: annual 3P-MACE rates of 2.5–2.6%/year have been reported in real-world T2DM cohorts (e.g. Martín-Enguix, 2025), rising to 6.5%/year in Spanish patients with DM and established coronary syndrome (Mateos de la Haba, 2022, CICCOR study). Our assumption sits within this range, reflecting our mixed HR/VHR population (71% VHR, 39.2% ASCVD, 37.1% DM, 39.2% with atherosclerotic cardiovascular disease). We have updated the primary analysis, accordingly, as agreed with the clinical expert co-authors. More fundamentally, the baseline rate does not affect the relative comparison between strategies, as the CTTC-derived RRR applies proportionally in both arms. The sensitivity analysis across 2.0%–4.0% (S7 Table) confirms that all directional conclusions are fully robust regardless of the rate assumed.

Comment 2: "Perform and report sensitivity analyses using alternative baselines (e.g., 2%, 2.5%, 4%)."

Response: We thank the Reviewer for this suggestion, which we have fully implemented. We have conducted a linear sensitivity analysis of prevented 4P-MACE events across five baseline annual event rates: 2.0%, 2.5%, 3.0% (primary analysis), 3.5%, and 4.0%. Results are reported in the new Supplementary Table S7. The results confirm that the directional conclusions are robust across the entire range cited by the Reviewer: regardless of the baseline rate assumed, the BA-inclusive strategy consistently prevents fewer MACE than direct PCSK9i escalation (by 0.20–0.46 events per 100 patients/year), and this difference scales proportionally with the assumed rate. The absolute difference between strategies remains modest across all scenarios, further supporting the Discussion framing that the clinical trade-off is small relative to the substantial cost savings.

Comment 3: "Clarify whether the RRR (21% per 1 mmol/L LDL-C reduction from CTTC) is applied to the absolute LDL-C reduction from the post-EZE level (or from untreated baseline) and how 'prevented events' are computed differentially between BA-inclusive vs. BA-free arms."

Response: We thank the Reviewer for requesting this important clarification, which we have now addressed explicitly in the Methods section.

The 21% RRR per 1 mmol/L (38.67 mg/dL) LDL-C reduction (CTTC, Lancet 2010) is applied sequentially at each step of the simulation, consistently from the LDL-C level at the start of each treatment step (i.e., not from the untreated baseline), in a manner methodologically consistent with Katzmann et al. (2022). Specifically, prevented events are computed across three sequential steps, both arms sharing Step 1:

• Step 1, EZE step: The RRR is applied to the mean LDL-C reduction achieved by EZE simulation across the full cohort (N=34,967). For patients not yet on EZE (n=31,501), this reflects the reduction from their baseline LDL-C to the post-EZE simulated level; for patients already on EZE (n=3,466), no additional LDL-C reduction is attributed to EZE. The effective mean LDL-C reduction across the full cohort is used to compute events prevented at this step. This ensures that EZE-mediated MACE prevention is captured for all newly EZE-simulated patients.

• Step 2, BA step: For patients entering the BA simulation and achieving LDL-C control (n=11,167; 38.3% of BA entrants), the RRR is applied to the LDL-C reduction from the post-EZE level to the post-BA level.

• Step 3, E/A or INC step: For patients not controlled at BA (n=17,977), the BA LDL-C effect is reversed to the post-EZE level before E/A or INC simulation, and the RRR reflects only the E/A or INC reduction from that post-EZE level. In the Comparator arm, the RRR is applied to the E/A or INC reduction from the post-EZE level for all BA-eligible patients (n=29,144).

The differential in prevented events between arms arises because direct E/A or INC escalation achieves a greater absolute LDL-C reduction than BA followed by E/A or INC (since the latter group starts PCSK9i from a lower LDL-C baseline due to partial BA response in controlled patients, but the non-controlled patients start from the same post-EZE level in both arms), resulting in more prevented MACE in the Comparator arm, a trade-off that comes at higher cost.

We have updated this section of the manuscript to clarify readers.

Comment 4: "Explicitly state in Limitations that the small difference (0.3–0.4 events per 100 patients/year) is sensitive to these assumptions and that long-term modeling (beyond 1 year) with discounting would be valuable for future work."

Response: We thank the Reviewer and have added the following to the Limitations section:

"The estimated difference in prevented 4P-MACE events between BA-inclusive and BA-free strategies (0.2–0.5 events per 100 patients per year across the range of plausible baseline incidence rates tested in sensitivity analyses) is modest and sensitive to the assumed baseline annual event rate and the CTTC-derived RRR per mmol/L LDL-C reduction. Furthermore, the present model is limited to a 1-year time horizon. Long-term modelling incorporating time-discounting, treatment discontinuation, and event-free survival beyond 12 months would provide a more complete health-economic picture and is recommended for future work."

Comment 5: "Explicitly note that actual NHS acquisition costs (often discounted via tenders) differ, especially since PCSK9i/inclisiran are fully reimbursed while bempedoic acid reimbursement is more restricted."

Response: We thank the Reviewer for this observation. We have added the following to the Limitations section:

"Cost estimates are based on official list prices published by the Spanish Ministry of Health, as recommended by the CAPF/GENESIS pharmacoeconomic evaluation guidelines. In practice, the Spanish NHS may acquire drugs at negotiated prices below list, and BA, E/A, and INC are all reimbursed under restricted indications and special funding conditions, such as expenditure ceilings, which affect the final cost to the NHS. Furthermore, the simulation did not account for current Spanish reimbursement criteria for PCSK9i, which restrict eligibility to patients with LDL-C >100 mg/dL, well above ESC guideline targets, meaning the benefit of BA would likely be greater if these thresholds were applied, as BA would represent the only escalation option for many patients after statin and EZE."

Comment 6: "The savings estimate (~€1,174–1,679 per patient/year) therefore reflects a payer perspective using list prices; real-world NHS savings may be higher (or lower) depending on contracts."

Response: We thank the Reviewer for this observation, which is closely related to Comment 5. We confirm and have now explicitly stated in the Methods (Budget Impact section) that the cost analysis reflects a retail list price perspective, consistent with Spanish pharmacoeconomic evaluation guidelines. We have added:

"All costs reflect official retail prices and represent a payer perspective based on list prices. Real-world NHS acquisition costs may differ due to negotiated discounts or risk-sharing agreements, and the present estimates should be interpreted accordingly."

Comment 7: "For inclisiran, confirm use of only the maintenance-year cost (€4,696) and add a sensitivity analysis using first-year cost (€7,045)."

Response: We confirm that only the maintenance-year cost (€4,696) was used in the base-case analysis. This is a deliberate methodological choice to avoid overestimating savings in the first year and to align with prior simulation studies. We have added a new sensitivity analysis (Supplementary Table S8) using the first-year inclisiran cost (€7,045). Results confirm that using first-year inclisiran costs would further increase the estimated savings from the BA-inclusive strategy (-30.7% vs -27.2%). These percentages reflect the relative reduction in annual treatment cost per patient across the full simulated cohort (N=34,967), weighted by the proportion of patients reaching each treatment step — that is, they are not limited to patients who ultimately received INC, but account for the entire treatment sequence including those controlled at EZE or BA and therefore not escalated to INC. As a result, the weighted average cost per patient is lower than the per-patient INC cost, and the relative savings from incorporating BA before INC reflect the full budget impact across all simulated patients.

Comment 8: "Limitations Section – Expand Slightly. Add: (a) geographic coverage limited to three regions in the IQVIA EMR (~3% of Spanish population, though demographically representative); (b) no modeling of adherence, discontinuation, or titration delays (real-world persistence with oral BA may exceed injectables); (c) uniform application of NMA efficacy across subgroups (no interaction testing by baseline LDL-C, statin intensity, or risk category)."

Response: We have expanded the Limitations section to address each point explicitly:

(a) "The IQVIA EMR database covers three health regions in Spain, representing approximately 3% of the national population (~1.2 million patients). While prior studies have confirmed its demographic representativeness of the broader Spanish population, generalizability to all

Attachment Submitted filename: Response to Reviewers.docx

# Peer review: Decision Letter 1

**DOI:** 10.1371/journal.pone.0353193.r003

**Authors:** Paolo Magni

21 Jun 2026

LDL-C target attainment and treatment costs in high-risk and very-high-risk patients with or without bempedoic acid: a Spanish cohort simulation

PONE-D-25-64496R1

Dear Dr. Juan Cosin Sales,

We’re pleased to inform you that your manuscript has been judged scientifically suitable for publication and will be formally accepted for publication once it meets all outstanding technical requirements.

Within one week, you’ll receive an e-mail detailing the required amendments. When these have been addressed, you’ll receive a formal acceptance letter and your manuscript will be scheduled for publication.

An invoice will be generated when your article is formally accepted. Please note, if your institution has a publishing partnership with PLOS and your article meets the relevant criteria, all or part of your publication costs will be covered. Please make sure your user information is up-to-date by logging into Editorial Manager at Editorial Manager® and clicking the ‘Update My Information' link at the top of the page. For questions related to billing, please contact billing support.

If your institution or institutions have a press office, please notify them about your upcoming paper to help maximize its impact. If they’ll be preparing press materials, please inform our press team as soon as possible -- no later than 48 hours after receiving the formal acceptance. Your manuscript will remain under strict press embargo until 2 pm Eastern Time on the date of publication. For more information, please contact onepress@plos.org.

Kind regards,

Paolo Magni

Academic Editor

PLOS One

Additional Editor Comments (optional):

Paper improved. Reviewers' comments addressed in full.

Reviewers' comments:

Reviewer's Responses to Questions

**Comments to the Author**

Reviewer #1: All comments have been addressed

**********

2. Is the manuscript technically sound, and do the data support the conclusions?

Reviewer #1: Yes

**********

3. Has the statistical analysis been performed appropriately and rigorously?

Reviewer #1: Yes

**********

4. Have the authors made all data underlying the findings in their manuscript fully available?

The PLOS Data policy

Reviewer #1: Yes

**********

5. Is the manuscript presented in an intelligible fashion and written in standard English?

Reviewer #1: Yes

**********

Reviewer #1: All of my comments have been addressed. I congratulate authors for this amazing work. I have not further suggestions.

**********

what does this mean?). If published, this will include your full peer review and any attached files.

If you choose “no”, your identity will remain anonymous but your review may still be made public.

**Do you want your identity to be public for this peer review?**  For information about this choice, including consent withdrawal, please see our Privacy Policy

Reviewer #1: No

**********

# Editor report: Acceptance letter

**DOI:** 10.1371/journal.pone.0353193.r004

**Authors:** Paolo Magni

PONE-D-25-64496R1

PLOS One

Dear Dr. Cosin-Sales,

I'm pleased to inform you that your manuscript has been deemed suitable for publication in PLOS One. Congratulations! Your manuscript is now being handed over to our production team.

At this stage, our production department will prepare your paper for publication. This includes ensuring the following:

* All references, tables, and figures are properly cited

* All relevant supporting information is included in the manuscript submission,

* There are no issues that prevent the paper from being properly typeset

You will receive further instructions from the production team, including instructions on how to review your proof when it is ready. Please keep in mind that we are working through a large volume of accepted articles, so please give us a few days to review your paper and let you know the next and final steps.

Lastly, if your institution or institutions have a press office, please let them know about your upcoming paper now to help maximize its impact. If they'll be preparing press materials, please inform our press team within the next 48 hours. Your manuscript will remain under strict press embargo until 2 pm Eastern Time on the date of publication. For more information, please contact onepress@plos.org.

You will receive an invoice from PLOS for your publication fee after your manuscript has reached the completed accept phase. If you receive an email requesting payment before acceptance or for any other service, this may be a phishing scheme. Learn how to identify phishing emails and protect your accounts at https://explore.plos.org/phishing.

If we can help with anything else, please email us at customercare@plos.org.

Thank you for submitting your work to PLOS ONE and supporting open access.

Kind regards,

PLOS ONE Editorial Office Staff

on behalf of

Prof. Paolo Magni

Academic Editor

PLOS One
